# DNA Double Strand Break Repair Is Important for the Longevity of Primed Seeds

**DOI:** 10.1111/pce.70142

**Published:** 2025-08-21

**Authors:** Wanda M. Waterworth, Dapeng Wang, Lerissa S. Dsilva, Christopher E. West

**Affiliations:** ^1^ Faculty of Biological Sciences University of Leeds Leeds UK; ^2^ Shandong Key Laboratory of Intelligent Oil & Gas Industrial Software, Qingdao Institute of Software, College of Computer Science and Technology China University of Petroleum (East China) Qingdao China; ^3^ National Heart and Lung Institute Imperial College London London UK

**Keywords:** DNA repair, hypoxia, osmopriming, PCD, priming, proteosome, RNAseq, seed biology, transcriptomics

## Abstract

Germination of many crop species is improved by priming, which facilitates pre‐germinative metabolism through controlled hydration. However, priming is often associated with reduced seed longevity. Here, a screen of *Arabidopsis thaliana* DNA repair mutants identified *dna ligase 6* and *dna ligase 4* (*lig6lig4*) seeds as most sensitive to ageing of primed seed. Genetic analysis of wild type and *lig6lig4* mutants provided mechanistic insight into the link between DNA double strand break (DSB) repair and longevity of primed seeds. RNAseq analysis of naturally aged seeds demonstrated that, while the transcriptome changes in primed aged seeds mirrors the enhancement of germination, priming significantly activated the transcriptional response to chromosomal breaks and, in *lig6lig4* mutant seed, greatly exacerbated programmed cell death. These results revealed that DSB repair is an important factor in promoting longevity of primed seed, further supported by the improved longevity of primed seeds with enhanced expression of *LIG6*. Collectively our findings establish the genetic requirement for LIG6 in longevity of primed seed and indicate that the reduced longevity of primed seeds is mitigated by DSB repair activities. These results provide insight into the molecular basis of the reduced longevity of primed seed, important for sustainable crop production under changing climates.

## Summary Statement

Seed priming is widely used to improve germination of crop species but often leads to loss of seed storability. Here we show pathways for the repair of DNA double strand breaks are required to maintain the longevity of primed seeds, promoting germination and minimising cell death.

## 1 Introduction

Seed germination performance is central to agriculture and plant survival in the natural environment. High seed vigour, defined as rapid, uniform germination and robust seedling establishment, tolerant of adverse environmental conditions, is a major determinant of successful crop yields (Finch‐Savage and Bassel 2016; Rehmani et al. 2023). Low quality seed negatively impacts on final yield through reduced emergence, slower and weaker seedling establishment and reduced harvesting efficiency arising from non‐uniformity of crop growth. Reduced cellular maintenance in the dry seed and cycle(s) of desiccation and rehydration are associated with severe deterioration of cellular membranes, proteins and DNA in the seed (Nadarajan et al. 2023). Germination is initiated upon seed hydration (imbibition) and culminates in emergence of the radicle (young root) through the seed coat (Bewley 1997). The capacity to withstand or repair cellular damage is crucial to germination vigour and seed viability and loss of seed vigour is associated with an extended period of repair (Waterworth et al. 2016; Waterworth and West 2023). DNA damage accumulated in the dry seed must be repaired before initiation of cell cycle to minimise growth inhibition and mutation of genetic information. Chromosomal breaks (DNA double strand breaks (DSBs)) are a particularly cytotoxic forms of DNA damage, potentially resulting in chromosome fragmentation, loss of genetic information and cell death. Genetic analysis of *Arabidopsis thaliana* (Arabidopsis) mutants deficient in the DSB repair factors DNA LIGASE 4 (LIG4) and DNA LIGASE 6 (LIG6) established the importance of DSB end‐joining pathways for seed longevity (Waterworth et al. 2010, 2022).

Several crop species, including high value vegetable seeds, are routinely improved by priming, a pre‐germinative seed treatment in which controlled hydration increases the speed of germination and enhances field emergence (Heydecker et al. 1973). Controlled hydration using an osmoticum (osmopriming), partial hydration or limited time of hydration (hydropriming), allows cellular repair processes to proceed before drying back before completion of germination (Heydecker et al. 1973; McDonald 1999). Priming reverses the lag period to germination in low vigour seeds and promotes uniformity of germination and stress tolerant growth of emerging seedlings. Seedling field emergence for many commercial species can be increased 5%–10% by priming (Finch‐Savage and Bassel 2016). However, priming can result in a significant reduction in seed longevity as the loss of viability over time is accelerated, resulting in substantial economic losses in crop species (Tarquis and Bradford 1992; Dekkers et al. 2015). The molecular basis for this loss of storability remains unknown, although over‐priming, where germination is allow to progress to the initiation of DNA replication, was associated with reduced viability in tomato (vanPijlen et al. 1996). Previous studies have suggested that seed priming treatments improve germination vigour by enhancing the innate seed stress response through exposure to stress conditions during the priming process. This can result in seeds with improved resilience upon drying back, for example through increased levels of antioxidants (Pagano et al. 2022). Analysis of factors associated with reduced longevity of primed seeds identified differences in brassinosteroid (BR) signalling genes and cell wall modification factors in priming‐sensitive Arabidopsis ecotypes (Sano et al. 2017). Recently, RNAseq analysis of hydroprimed Arabidopsis seeds, in which access to water was time‐limited, reported decreased ribosome association of stress‐responsive transcripts using polysome profiling, providing an explanation for the increased stress‐resistance of primed seeds (Gran et al. 2025). Furthermore, increased expression of base excision repair enzymes has been reported in primed seeds (Forti et al. 2020).

To establish the molecular basis by which priming reduces seed longevity, we screened mutants in the main plant DNA repair pathways, finding that the repair of DSBs by DNA ligases was most critical to the lifespan of primed seeds. Therefore, we analysed the transcriptional profiles associated with priming naturally aged wild type and *lig6lig4* mutants compared to control high quality seed lots. RNAseq revealed that the vigour improvement exhibited by primed seeds was reflected at the transcriptional level, with priming largely removing the response to seed ageing. However, priming significantly activated the transcriptional response to chromosomal breaks in wild type and DSB repair mutant seeds and resulted in extensive programmed cell death (PCD) in *lig6lig4* mutants. These results provide a mechanistic explanation for the requirement for DNA ligases in primed seeds and highlights DSB repair as an important factor in both seed ageing and the reduced longevity of primed seed. Confirmation of these findings was provided by analysis of transgenic seed lots with elevated levels *LIG6* expression, which displayed an enhanced resistance to accelerated ageing. Collectively our findings establish the genetic requirement for DSB repair in seed priming and the function of DNA ligases to promote the longevity of primed seeds.

## 2 Materials and Methods

### 2.1 Plant Material and Growth Conditions

*Arabidopsis* plants were raised in growth chambers under constant humidity (30%), with 16‐h light and 8‐h dark cycles at 23°C. Plants were grown on half‐strength MS, 1% sucrose, 0.5 g l^−1^ 2‐(N‐morpholino)ethanesulfonic acid (MES) and 0.8% plant agar (Duchefa) pH 5.7 on 16 h:8 h light–dark cycles at 22°C. The following genotypes were obtained from the NASC and have been described previously: Col‐0, *arp1‐1* (AT2G41460, SALK_021478), *ercc1‐1* (AT3G05210, SALK_033397), *ku70‐1* (AT1G16970, SALK_123114), *atr‐2* (AT5G40820), *atm‐3* (AT3G48190, SALK_089805), *lig6‐1* (AT1G66730, SALK_079499), *lig4‐5* (AT5G57160, SALK_095962) and *xrcc2‐1* (AT5G64520, SALK_029106) (Waterworth et al. 2016, 2022). The *sog‐2* (AT1G25580) mutant was generated using CRISPR‐Cas9 mutagenesis (Waterworth et al. 2022). For each experimental replicate, seeds from all lines were harvested simultaneously and stored at 15°C and 15% humidity for 2 months to allow after‐ripening. Germination tests and accelerated ageing were performed according to published protocols (Waterworth et al. 2022). Accelerated ageing was performed at 35°C and 83% relative humidity by incubating seeds over saturated KCl in a sealed container. For natural ageing experiments *colo* and *lig6lig4* seed harvests dated from 2012 to 2020 were stored at ambient temperature and humidity as described previously (Waterworth and West 2023). Arabidopsis seeds were primed as previously described with −0.75 MPa PEG6000 for 48 h in the dark at 20°C before washing with dH_2_O to remove residual PEG. Seeds were then dried spread out thinly on white filter paper in a drying oven at 23°C in a thin layer in the dark for 3 days. After drying back, the weight of 100 primed *Arabidopsis* seeds was comparable to that of unprimed control seeds.

### 2.2 Plant Genotyping, Overexpression and Microscopy

Arabidopsis DNA extraction for PCR genotyping was performed by grinding plant tissue in shorty buffer (0.2 M Tris pH 9.0, 1% sodium dodecyl sulphate (SDS), 0.4 M LiCl, 25 mM ethylenediaminetetraacetic acid (EDTA)) in a 1.5 mL microfuge tube using a plastic micropestle. Cell debris was pelleted at 13 000*g* for 5 min and the supernatant mixed 1:1 with 100% isopropanol and DNA recovered by centrifugation and dissolved in TE buffer. Plant genotyping was performed by PCR (GoTaq, Promega) using primers designed by iSect software (signal.salk.edu/tdnaprimers.2.html) and insertion sites confirmed by sequence analysis (Genewiz). LIG6 overexpression was performed by cloning *LIG6* cDNA in pENTR1A using primers as listed in Supporting Information: Table S1 using Gibson assembly into BamHI XhoI digested vector. The cDNA was subcloned into pEarleyGate100 by Gateway cloning, providing plant expression under 35S. The construct was introduced into Col‐0 lines using floral dip transformation with Agrobacterium strain GV3101 and seeds were selected on media supplemented with hygromycin. Lines with elevated *LIG6* expression were identified by qRT‐PCR. Confocal microscopy was performed using a Zeiss LSM880 inverted microscope. PCD was analysed after staining with propidium iodide (10 mg/L). The *z*‐section containing the quiescent centre was used for quantification of cell death area using the microscope operating software (Zen, Zeiss).

### 2.3 Sequence Analysis

Total RNA was isolated from seeds ground in liquid nitrogen using an SV RNA isolation kit (Promega). Library preparation and paired‐end sequencing was conducted by Novogene. Quality assessment of FASTQ files was performed using FastQC (https://www.bioinformatics.babraham.ac.uk/projects/fastqc/), and adapter sequence was removed using Cutadapt (Martin 2011), followed by trimming and filtering of low‐quality reads using PRINSEQ (Schmieder and Edwards 2011). Processed reads were aligned to the *Arabidopsis thaliana* reference genome (TAIR10) from Ensembl Plants (release 50) (Howe et al. 2020) using the STAR aligner (Dobin et al. 2013). Alignment files were then processed with SAMtools (Li et al. 2009) for format conversion, sorting, and indexing. Only uniquely mapped reads were retained for downstream analysis. Gene‐level read counts were generated from the GTF annotation using the featureCounts() function in the Rsubread package (Liao et al. 2019). Differential expression analysis was performed using the DESeq. 2 package (Love et al. 2014) to identify significantly altered genes across experimental conditions. Confirmation of gene expression profiles was performed using qPCR with a CFX96 thermocycler and Ssofast SYBR green (BioRad) with normalisation to *ACTIN7*. Primer sequences are presented in Supporting Information: Table S1. Gene ontology analysis was performed using g:Profiler (https://biit.cs.ut.ee/gprofiler/gost). Gene set enrichment analysis was performed as described previously (Mootha et al. 2003; Subramanian et al. 2005) using available software (https://www.gsea-msigdb.org/gsea/index.jsp). Germination time series gene sets were generated using published microarray data (Nakabayashi et al. 2005; Winter et al. 2007; Bassel et al. 2008) by pairwise comparison of each timepoint with dry seeds using limma (Ritchie et al. 2015). The top 30 genes with highest positive or negative fold change in expression between time points (filtering out genes that are represented at earlier time points) were used to generate GMX files (Supporting Information: Table S2). RNAseq data for each treatment was compared to dry seeds and genes ranked by fold change for testing for enrichment against the GMX file to produce a normalised enrichment score (NES) quantifying the overrepresentation of the gene set in the upper or lower sections of the ranked differential expression list.

### 2.4 Statistical Analyses

Data were analysed using R (R_Core_Team 2025). Kolmogorov–Smirnov (KS) tests (*p* > 0.05) were used to test for normality, homogeneous variance was determined using Levene's test (*p* > 0.05). ANOVA with post hoc Tukey's tests was used for multiple comparisons and Kruskal–Wallis Test with Bonferroni correction used for non‐normal data. Boxplots display the interquartile range (IQR), with whiskers showing the data range within 1.5‐fold of the IQR.

## 3 Results

### 3.1 Genome Maintenance Pathways Important for Longevity of Primed Seeds

**Figure 1:**
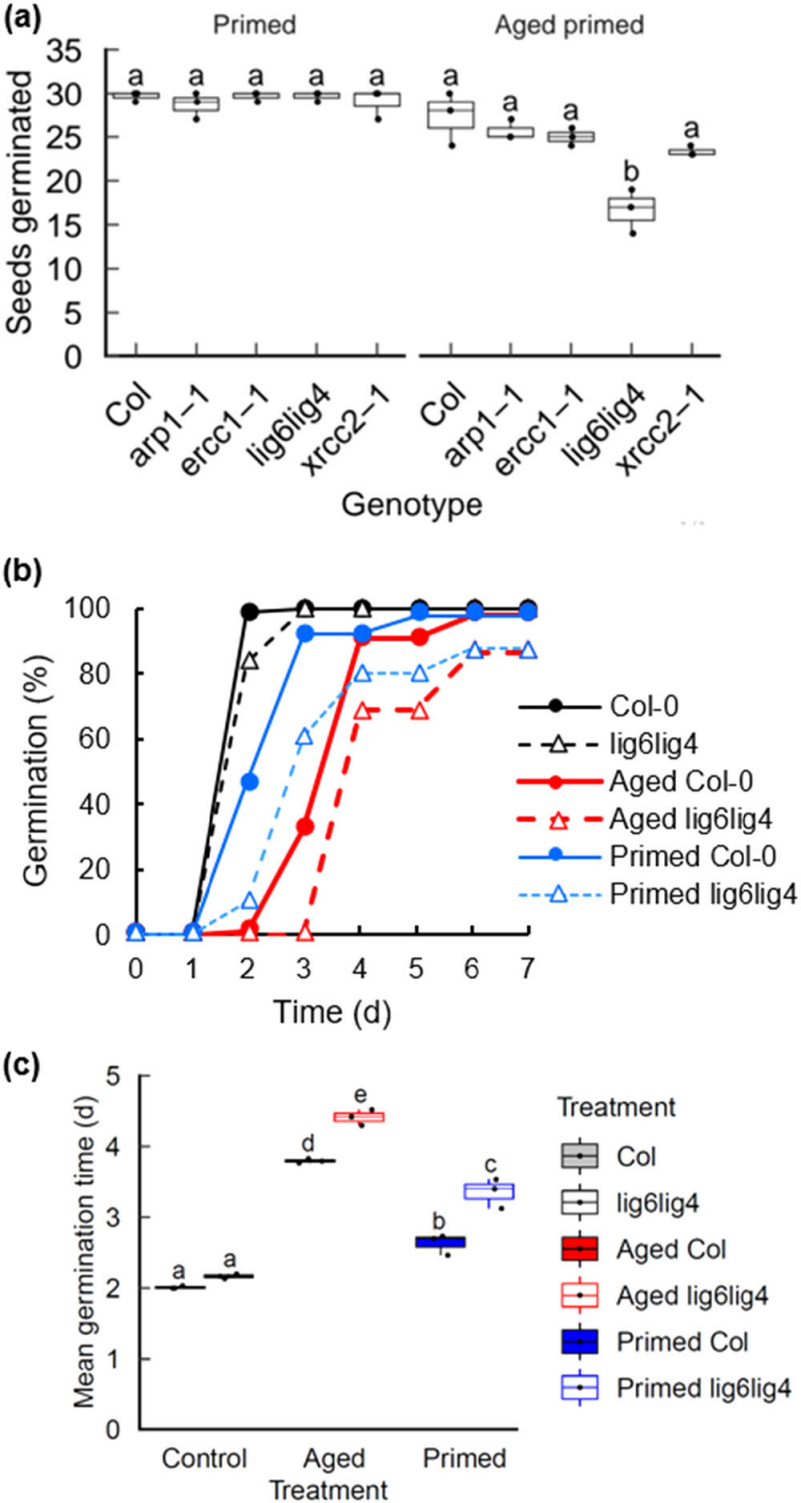
Germination analysis of primed wild type and DNA repair mutant seeds. Seeds were stratified at 4°C for 2 d before transfer to 23°C 16‐h day and scored for radicle emergence each day post‐stratification. (a) Seeds of WT and DNA repair mutant lines were primed for 48 h with −0.75 MPa PEG6000 before drying. Germination of primed Col‐0 and mutant primed seed was analysed before and after accelerated aging at 35°C and 83% RH for 7 days. (b) Germination profiles of Col‐0 and *lig6lig4* mutant lines, showing control (unaged) seeds, seed lots naturally aged for 10 years and aged seed lots subjected to priming. Data for control and aged seeds was previously published in Waterworth and West (2023). (c) Mean germination time of Col‐0 and *lig6lig4* mutant lines. Letters denote homogeneous subsets (*p* < 0.01, ANOVA with Tukey correction). Error bars represent the SEM of the mean of three replicates of 30 seeds. Wild type: filled boxes; *lig6lig4* mutants: unfilled boxes. ANOVA, analysis of variance; RH, relative humidity; SEM, standard error of mean; WT, wild type. [Color figure can be viewed at wileyonlinelibrary.com]

To establish the role of DNA repair pathways in the storability of primed seeds, the longevity of mutant seed deficient in the main plant DNA repair pathways was analysed post‐priming. Repair factors investigated included the base excision repair gene *ARF1* and the nucleotide excision repair factor *ERCC1*. Chromosomal break repair factors included *XRCC2* (homologous recombination) and the double *lig6lig4* mutant deficient in both NHEJ and alternative end‐joining (Waterworth et al. 2010). Wild type and mutant Arabidopsis seed were osmoprimed in PEG6000 for 48 h before drying and accelerated ageing (35°C and 83% relative humidity for 7 days), followed by evaluation of germination performance (Figure 1a). Mean germination time (MGT) in the wild type and DNA repair deficient mutant lines was decreased by priming, consistent with advancement of germination in all genetic backgrounds. However, primed *lig6lig4* seeds showed a marked decrease in viability after seed ageing compared to other mutant lines, implicating DSB repair as an important mechanism for maintenance of seed longevity after priming. Seed ageing can result in the appearance of abnormal seedlings which can be classified according to published standards (www.seedtest.org). The priming and accelerated ageing regimes used here produced no significant increase in the frequency of abnormal seedlings (Supporting Information: Figure S1).

### 3.2 Priming of Naturally Aged Wild Type and DSB Repair‐Deficient *lig6lig4* Seed Lots

The aim of this study was to determine the seed stress responses associated with osmopriming, specifically to determine the genetic requirement for DNA ligases in primed seed and to identify evidence of chromosomal break signalling pathways. Previously we demonstrated that *lig6lig4* DSB repair mutants displayed hyper‐sensitivity to accelerated ageing (Waterworth et al. 2010) and more recently confirmed this result with naturally aged seeds (Waterworth and West 2023). These studies established DSB end‐joining as a determinant of longevity in seed ageing. LIG4 catalyses the final DNA end joining step in the canonical NHEJ pathway, conserved across eukaryotes, whilst LIG6 participates in an alternative NHEJ (Alt‐NHEJ) back‐up pathway, with loss of both factors progressively reducing germination vigour (Waterworth et al. 2010). Here, to further investigate the link between DSB repair and priming, we compared the effects of osmopriming the previously characterised naturally aged Arabidopsis seed lots (produced in 2012) of wild type (Col‐0) and *lig6lig4* double mutants (Waterworth and West 2023), using unaged seed lots (2022) as controls. The aged seed lots were primed with −0.75 MPa PEG6000 for 48 h before washing and drying. Priming significantly increased the germination vigour of aged seeds of both wild type and *lig6lig4* mutant lines, with a markedly decreased mean germination time after priming, consistent with priming advancing germination of aged seeds (Figure 1b,c). For all treatments the *lig6lig4* mutant line displayed lower germination vigour than wild type.

**Figure 2:**
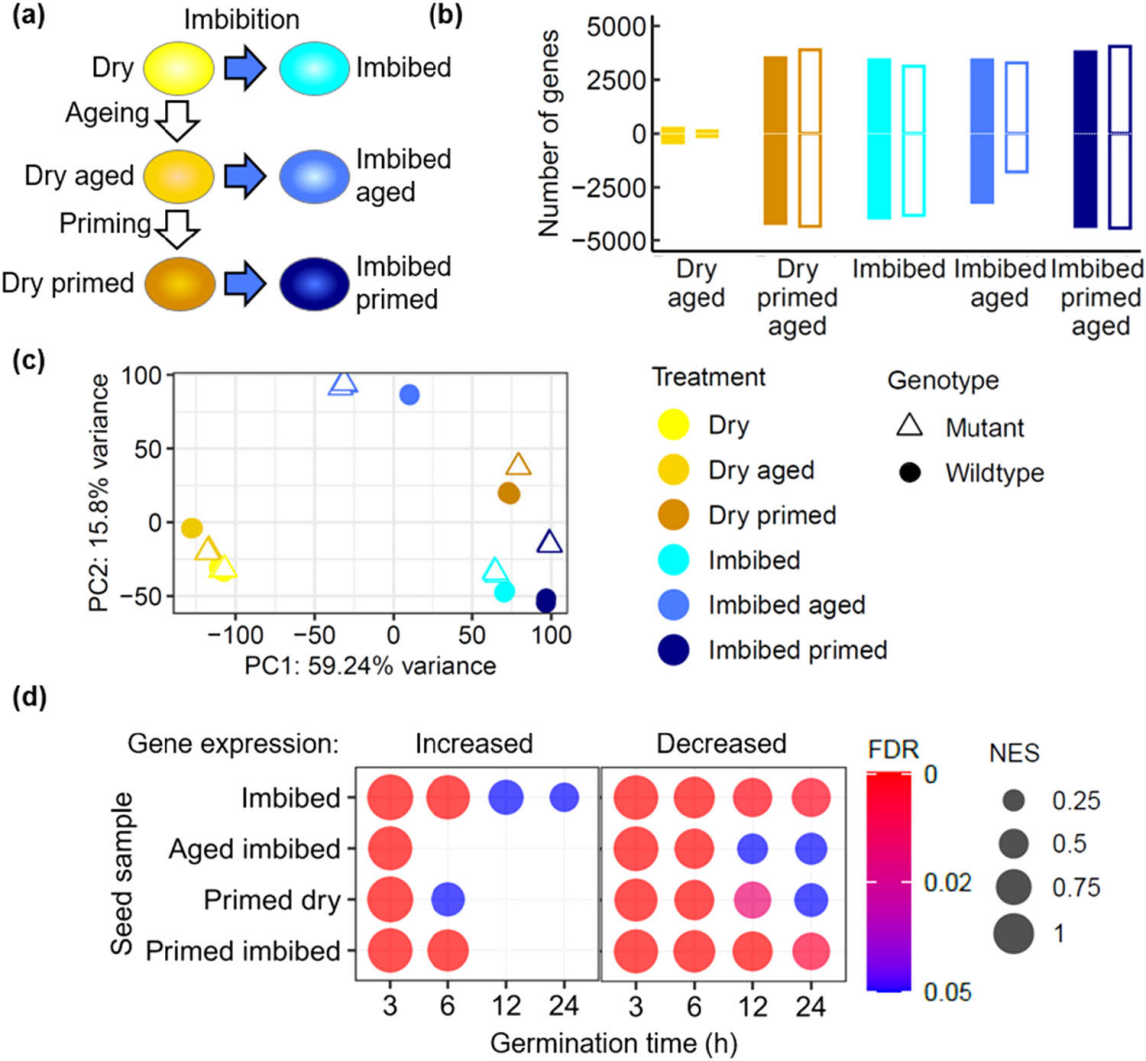
Analysis of gene expression during priming of naturally aged *lig6lig4* mutant seeds. (a) Outline of samples used in the analysis. Each treatment was repeated in triplicate for both wild type and *lig6lig4* mutant lines. (b) Numbers of differentially expressed genes relative to dry control Col‐0 seeds for each of the treatments. (c) Principal component analysis of gene expression levels in each of the samples of wild type (Col‐0) and mutant (*lig6lig4*) including dry seeds (control and naturally aged seed before and after priming) and 6 h imbibed seeds (control and naturally aged seed before and after priming). (d) Expression of genes associated with germination progression in each sample was assayed using gene set enrichment analysis in Col‐0. Normalised enrichment score (NES) is plotted as point size and the adjusted *p*‐value (false discovery rate) is plotted by colour. Wild type: filled symbols; *lig6lig4* mutants: unfilled symbols. [Color figure can be viewed at wileyonlinelibrary.com]

Transcriptomic analysis of wild type and *lig6lig4* mutant seed lots was conducted to determine the gene expression patterns associated with the loss of germination vigour after seed ageing and recovery after seed priming. For each of the three treatments (control, aged and primed aged seeds), both dry seeds and seeds imbibed for 6 h were analysed, producing six treatments for each genotype (Col‐0 and *lig6lig4*) in three replicates (Figure 2a). The 6 h imbibition time point was selected for analysis as the transcriptional DNA damage response is activated early in Arabidopsis seed germination, around the reinitiation of cellular metabolism upon rehydration and peaks at 6 h (Waterworth et al. 2010). Principal component analysis identified small differences between Col‐0 and *lig6lig4* mutants. Most variation in expression was observed upon seed imbibition and after priming (PC1, Figure 2b,c). Seed ageing resulted in significant differences to control seeds in RNA expression profile upon seed imbibition (Figure 2c: PC2). Notably, priming was able to partially reverse this ageing‐related profile, such that the subsequent imbibition of primed seeds resulted in gene expression profiles similar to those of high quality, unaged seed lots. This demonstrates that the beneficial effects of seed priming on germination vigour are reflected at the transcriptional level, whereby priming alters the transcriptional profile of imbibed aged seeds to one more comparable to imbibed unaged seeds (Figure 2). The large changes in gene expression in imbibed seeds (Figure 2b) was previously shown to be a combination of the turnover of transcripts associated with seed maturation together with a rapid *de novo* synthesis of genes involved in germination (Nakabayashi et al. 2005). The transcriptional profiles associated with progression through germination was assessed using gene set enrichment analysis (GSEA) (Subramanian et al. 2005). Sets of genes displaying high expression at specific time points in germination were identified using a previously published time course of gene expression 0‐24 h after the onset of Arabidopsis seed imbibition (Nakabayashi et al. 2005) (Supporting Information: Table S2). Here, analysis of differentially expressed genes in imbibed Col‐0, relative to dry seeds, found enrichment of 3 h imbibition genes in all samples (Figure 2d). However, 6 h imbibition genes were only identified in imbibed unaged and imbibed primed seed samples and were not significantly enriched in imbibed aged seeds (Figure 2d). This is consistent with the delayed germination of aged seeds, reflected in a delayed profile of gene expression after seed imbibition. Similarly, analysis of genes that display reduced transcript levels following seed imbibition found evidence of delayed mRNA turnover in aged seeds which was largely restored by priming treatments (Figure 2d). Taken together, this data indicates that naturally aged seeds displayed delayed germination that is also reflected at the transcriptional level, but also reveals specific transcriptional responses to seed ageing, distinct from the normal imbibition transcriptional programme. Furthermore, seed priming reverses the effects of seed ageing, restoring transcriptional profiles closer to those of unaged, control seed lots.

### 3.3 Transcriptional Responses to Seed Ageing

**Figure 3:**
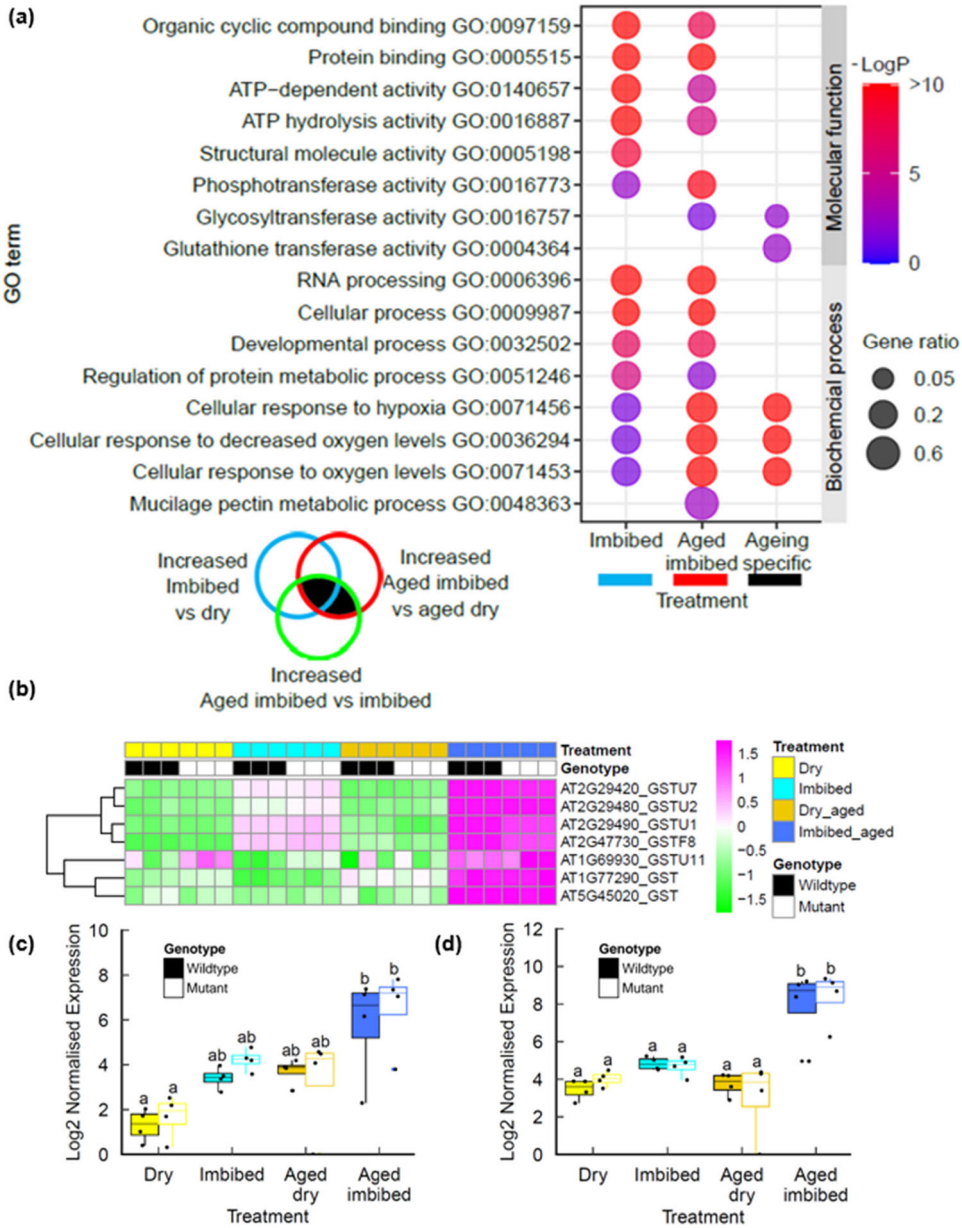
Transcriptional profiles of seed ageing in naturally aged seeds. (a) Ontology enrichment in genes differentially expressed in aged Col‐0 seeds. (b) Heatmap of Glutathione S transferase genes in naturally aged Col‐0 and *lig6lig4* mutant seeds. (c) qPCR of *GSTU2* expression in seeds. (d) qPCR of *GSTU7* expression in seeds. Letters denote homogeneous subsets (*p* < 0.01, ANOVA with Tukey correction). Wild type: filled boxes; *lig6lig4* mutants: unfilled boxes. ANOVA, analysis of variance; qPCR, quantitative polymerase chain reaction. [Color figure can be viewed at wileyonlinelibrary.com]

Seed longevity is complex quantitative agriculture trait which is determined by the interaction of multiple genetic and environmental factors (Zinsmeister et al. 2020). Previous studies have analysed transcriptional responses to seed ageing in pea, rice and Arabidopsis employing accelerated ageing protocols widely used to simulate natural seed deterioration (Chen et al. 2013; Wang et al. 2022; Taylor et al. 2023). However, the biochemical mechanisms of seed ageing under conventional storage conditions may differ from those of accelerated ageing methodologies that use elevated temperature and humidity (Hay et al. 2019). Here we analysed the transcriptomes of Arabidopsis seeds naturally aged over a 10 year period in ambient, dry‐storage conditions, which were previously reported to display a two‐fold reduction in germination vigour (*p* < 0.001) but no significant change in viability (*p* > 0.05) (Figure 1b,c; Waterworth and West 2023). RNAseq analysis revealed that dry seeds displayed relatively few differences in gene expression between aged and control seed lots (Figure 2b and Supporting Information: Table S3), with genes with higher expression in aged seeds enriched in oxidoreductase activity and reduced expression of ribosomal protein genes (Supporting Information: Figures S2, 3). Imbibition of control (unaged) seeds or aged seeds resulted in large changes in gene expression (Figure 2b; Supporting Information: Tables S4 and S5). Genes with increased expression in control unaged seeds at 6 h imbibition were enriched in translation and metabolic factors, reflecting the rapid resumption of cellular activity on imbibition (Liew et al. 2024; Gran et al. 2025), whereas aged seeds displayed distinct changes in gene expression (Figure 3a, Supporting Information: Figures S4, 5). In both control and aged seed lots, decreased expression of photosynthesis transcripts revealed turnover of transcripts remaining after seed maturation (Supporting Information: Figures S6, 7). The transcriptional differences between aged and unaged Col‐0 seeds were much larger after imbibition compared to differences in the dry seed, with over 6000 genes differentially expressed between imbibed aged seeds and imbibed controls (Supporting Information: Table S6 and Figures S8, 9). The transcriptional response associated with seed ageing was analysed by identifying those genes with increased expression after imbibition of aged seeds which displayed significantly higher expression levels than imbibed control seeds, referred to as ‘ageing‐specific’ in Figure 3a. These genes were enriched in hypoxia related transcripts (GO term 0071456, Supporting Information: Figures S10, 11). In addition to hypoxia‐related genes, wild type imbibed aged seeds displayed significant enrichment in glutathione transferase (GST) gene expression (Figure 3a), which was also observed in the *lig6lig4* mutant line (Figure 3b). The increased expression of GST genes in response to ageing was confirmed by qPCR (Figure 3c) and is in line with a previous study reporting increased *GSTU22* expression in aged seeds (Kimura and Nambara 2010).

### 3.4 Osmopriming Is Associated With Elevated Expression of Proteasome Components

**Figure 4:**
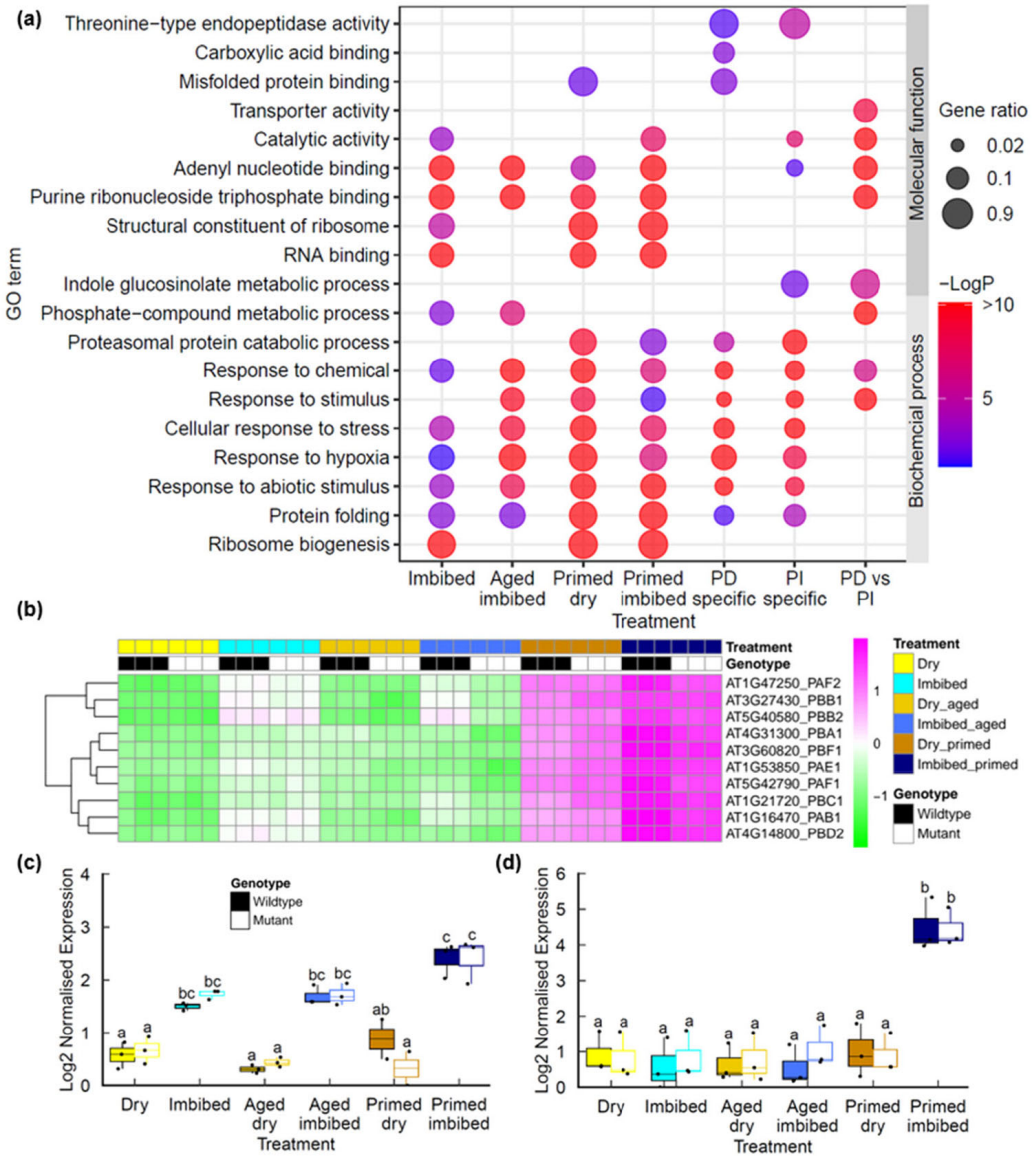
Transcriptional profiles of seed priming of naturally aged seeds. (a) Ontology enrichment in genes differentially expressed in primed Col‐0 seeds. (b) Heatmap of endopeptidase genes in naturally aged Col‐0 and *lig6lig4* mutant seeds. (c) qPCR of *PDD1* expression in seeds. (d) qPCR of *PDE2* expression in seeds. Letters denote homogeneous subsets (*p* < 0.01, ANOVA with Tukey correction). Wild type: filled boxes; *lig6lig4* mutants: unfilled boxes. ANOVA, analysis of variance; qPCR, quantitative polymerase chain reaction. [Color figure can be viewed at wileyonlinelibrary.com]

Our findings indicated that the loss of germination vigour exhibited by the aged seed lots (i.e., slower and less synchronous germination) can be reversed by seed priming treatment (Figure 1b,c). Primed seed lots of many species display reduced longevity, although the molecular basis for this remains unclear (Fabrissin et al. 2021b; Gran et al. 2025). Work in our lab and others has shown the chromosomal breaks contribute to loss of germination vigour (Waterworth et al. 2022) and base damage has also been implicated (Macovei et al. 2011; Chen et al. 2012; Cordoba‐Canero et al. 2014; Macovei et al. 2017). Here we conducted RNAseq to determine the transcriptional changes associated with seed priming in wild type and DSB repair mutants to gain insight into the cellular stresses associated with the controlled hydration of the priming process and subsequent dry back/imbibition cycle. Priming resulted in large changes in gene expression in both the dry primed seed and imbibed seeds (Figure 2c and Supporting Information: Tables S7, 8). As with imbibition, osmopriming is associated with a large hypoxia response and increased expression of ribosome biogenesis factors (Figure 4a), which surprisingly contrasts with the reduced hypoxia response in hydroprimed seeds (Gran et al. 2025). Large differences are observed in the transcriptional profile of primed seeds compared to imbibed seeds, with significant increases in components of proteasomal protein catabolic processes in both primed dry and primed imbibed seeds (Figure 4a) which was confirmed by qPCR (Figure 4c,d). Proteasome components can be induced by a range of cellular stresses including drought and pathogen infection (Xu et al. 2024) and is involved in modulating ABA‐mediated signalling through degradation of the proteasome component PDE1 (Han et al. 2019).

### 3.5 Analysis of DSB Responses in Primed Wild Type and *lig6lig4* Mutant Seeds

**Figure 5:**
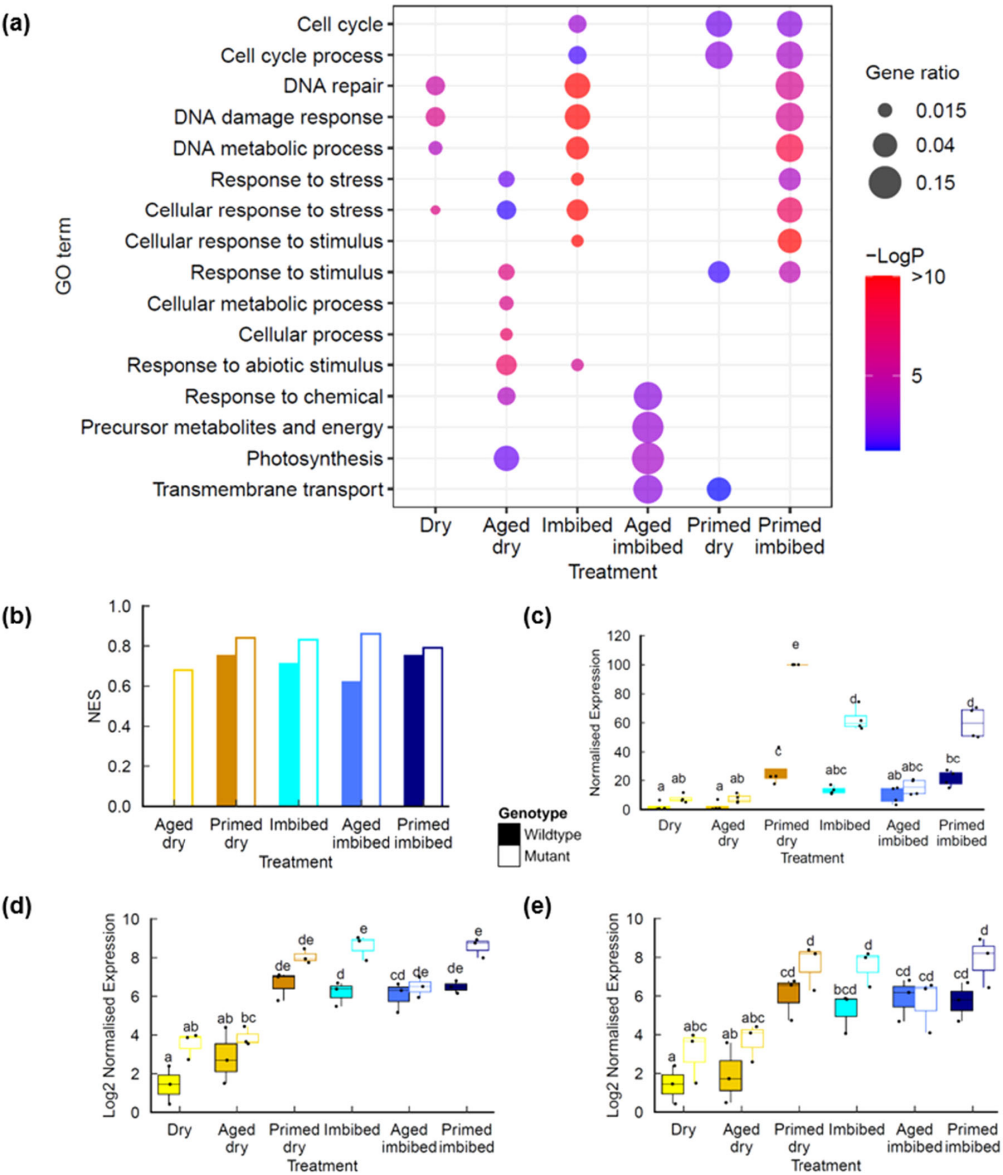
Transcriptional profiles in during priming of naturally aged Col‐0 and *lig6lig4* mutant seeds. (a) Ontology enrichment in genes differentially expressed in *lig6lig4* seeds relative to Col‐0. (b) DDR Gene set enrichment analysis comparing each treatment to Col‐0 dry seed expression values. NES values shown for samples that are display significant DDR gene expression enrichment (FDR < 0.01). (c) DDR gene expression in naturally aged Col‐0 and *lig6lig4* mutant seeds. Normalised expression of the top four inducible DDR genes (*BRCA1, XRI, PARP2, RAD51*) are shown (d) qPCR of RAD51 expression in seeds. (e) qPCR of *XRI* expression in seeds. Letters denote homogeneous subsets (*p* < 0.01, ANOVA with Tukey correction). Wild type: filled boxes; *lig6lig4* mutants: unfilled boxes. ANOVA, analysis of variance; DDR, DNA damage response; NES, normalized enrichment score; qPCR, quantitative polymerase chain reaction. [Color figure can be viewed at wileyonlinelibrary.com]

The *lig6lig4* mutant is deficient in the repair of DSBs and deficiency in both LIG6 and LIG4 results in an additive hypersensitivity to seed ageing, consistent with functions of each DNA ligase specific pathways of end joining (Waterworth et al. 2010). The decreased longevity of aged and primed *lig6lig4* mutant seeds (Figure 1) implicates the induction of chromosomal breaks as a factor in the reduced storability of primed seeds. Here, the transcriptional profile across the different seed treatments was compared between wild type and *lig6lig4* mutant seeds using gene ontology enrichment analysis of DEGs. The transcriptional DNA damage response (DDR) to chromosomal breaks is under control of Ataxia‐telangiectasia (ATM) (Culligan et al. 2006) and distinct to the induction of other repair pathways. The DSB‐DDR is observed early in seed imbibition, even in high vigour seeds (Waterworth et al. 2010; Sincinelli et al. 2025). Dry, imbibed and primed imbibed *lig6lig4* mutant seeds displayed elevated expression of genes associated with cellular response to stress, DNA repair and DNA metabolism, relative to Col‐0 (Figure 5a, Supporting Information: Table S9‐13). This is consistent with previous reports that *ku80* mutants displayed constitutive activation of the DDR (West et al. 2004). These GO categories are significantly upregulated in primed *lig6lig4* mutant after imbibition (Figure 5a), consistent with increased chromosomal breaks associated with priming. The transcriptional DNA damage response was further analysed using DSB‐DDR gene set enrichment analysis (GSEA) in pairwise comparisons of each treatment relative to dry Col‐0 seeds. The DDR gene set consisted of the top 22 DNA repair factors that were identified in the Arabidopsis response to gamma irradiation (Culligan et al. 2006). Both wild type and *lig6lig4* mutants display significant enrichment in DDR gene expression in imbibed seeds of unaged, aged and primed seeds (Figure 5b; FDR < 0.001), consistent with the presence of chromosomal breaks upon seed hydration and priming. DDR activation was further analysed in wild type and *lig6lig6* mutants, focussing on expression of the top four most inducible DNA repair genes (*RAD51*, *XRI1*, *PARP2* and *BRCA1*; (Culligan et al. 2006)). This further supported that increased genome stress is associated with priming, with an elevated DDR in primed seeds, especially in the *lig6lig4* mutant (Figure 5c). For example, expression of *RAD51*, a gene required for DSB repair, in wild type seeds is induced 17‐fold upon imbibition, but this value increases to ~30‐fold in primed seeds and 150‐fold in primed dry *lig6lig4* mutant seeds. The DDR activation identified in the RNAseq data was confirmed by qPCR (Figure 5d,e), highlighting the high levels of genome stress in primed seeds. The elevated DNA damage response in primed *lig6lig4* mutant seed is consistent with significant levels of chromosomal breaks and loss of longevity in primed DSB repair mutants (Figure 1a). This was further investigated though analysis of cell death in germinated seeds from different treatments.

### 3.6 Increased PCD in Primed *lig6lig4* DNA Ligase Mutant Seed

**Figure 6:**
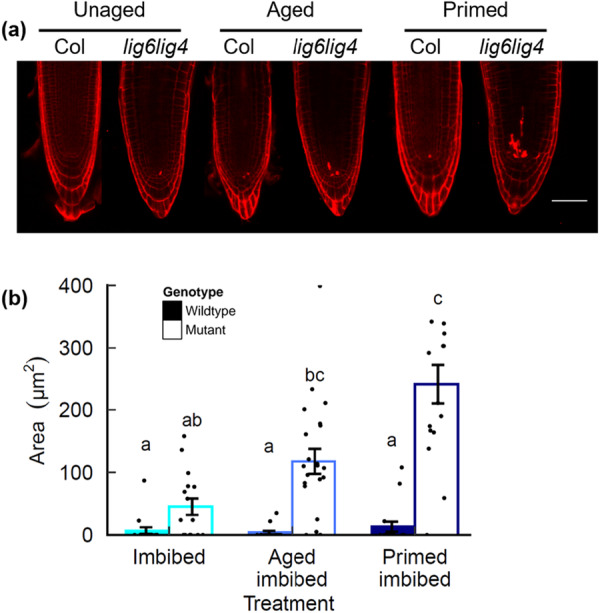
Programmed cell death (PCD) after priming of naturally aged *lig6lig4* mutant seed. Germination of unaged control, aged and primed aged wild type and *lig6lig4* mutant seed. Seeds were stratified at 4°C for 2 d before transfer to 23°C 16‐h day and scored for radicle emergence each day post‐stratification. PCD in wild‐type and DNA repair mutant seed was analysed by viability staining. (a) Confocal images of roots stained with propidium iodide. Bar is 50 μm (b) quantification of PI staining. PCD was quantified as the area of cell death in unaged control, aged and primed aged seeds. Control (unaged) seeds; Aged: seeds aged for 10 days at 35°C and 83% relative humidity (RH); Primed: aged seeds primed for 2 d. Significance groups are indicated by letters (*p* < 0.05, *n* > 10 per treatment, Kruskal–Wallis with Bonferroni post hoc correction for multiple tests) and data points represent PCD area of individual roots. Wild type: filled columns; *lig6lig4* mutants: unfilled columns. [Color figure can be viewed at wileyonlinelibrary.com]

A hallmark of the plant response to chromosomal breaks is SOG1‐dependent PCD in stem cell initials which eliminates cells with compromised genomes (Fulcher and Sablowski 2009). Previously, we identified that SOG1 induces PCD in the root apical meristem (RAM) in germinated aged seeds, indicative of increased genome damage (Waterworth et al. 2022). We therefore investigated the effects of priming on PCD in the RAM of wild type and *lig6lig4* mutant seeds using propidium iodide staining of recently harvested and naturally aged seed lots (2012) (Figure 6). We observed significantly higher incidence of cells undergoing PCD in aged *lig6lig4* seeds, as previously published (Waterworth et al. 2022). However, although germination vigour is significantly improved by priming treatment (Figure 1b), PCD is notably exacerbated in primed aged *lig6lig4* mutant seed (Figure 6b). This is indicative that seed priming is associated with marked increase in genome stress including DSBs, providing further support that the accumulation of DSBs contributes to the decreased longevity of primed seed.

### 3.7 *LIG6* Overexpression Improves Germination Vigour and Longevity of Primed Seed

**Figure 7:**
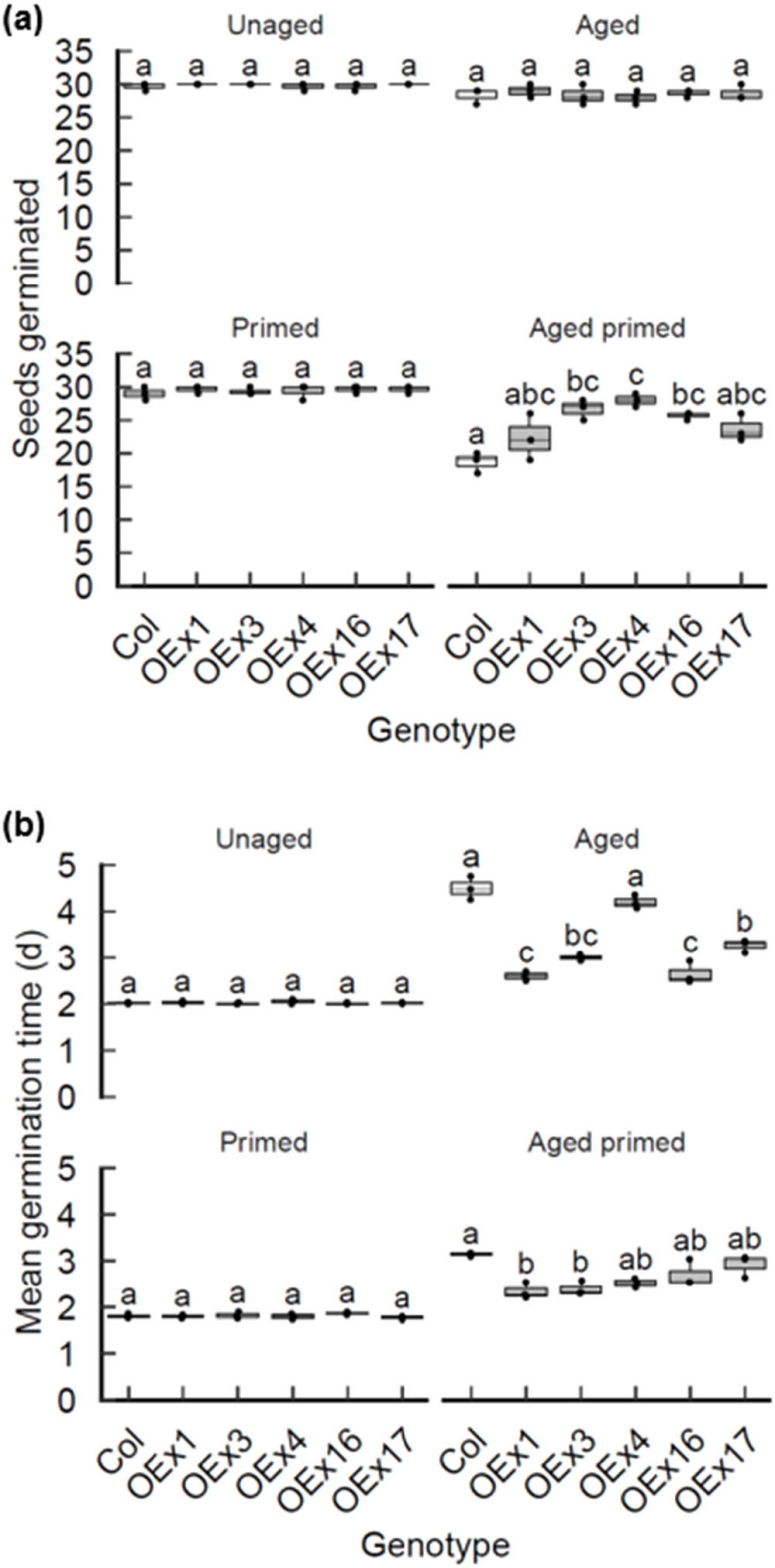
*LIG6* overexpression improves germination vigour and longevity of primed seed. Germination performance of transgenic *35S:LIG6* lines of Arabidopsis with increased expression of *LIG6* relative to wild type lines. (a) Viability of control wild type and *35S:LIG6* lines showing control, aged, primed and aged primed seed lots (aged after priming). (b) Mean germination time of control wild type and *35S:LIG6* lines showing control, aged, primed and aged primed seed lots. Accelerated ageing was performed at 35°C and 83% RH and priming was with −0.75 MPa PEG6000 for 48 h. Seeds were stratified at 4°C for 2 d before transfer to 23°C 16‐h day and scored for radicle emergence each day post‐stratification. Error bars represent the SEM of the mean of three replicates of 30 seeds. Data were analysed by ANOVA with Tukey's post hoc correction for multiple testing (*p* < 0.05) to compare genotypes within each treatment. ANOVA, analysis of variance; RH, relative humidity; SEM, standard error of mean.

Our data indicates that LIG4 and LIG6 are key determinants of seed longevity post‐priming, indicative of a requirement for genome maintenance in primed seeds. Deficiency in chromosomal break repair resulted in an elevated transcriptional DDR and increased incidence of PCD in the RAM in primed *lig4lig6* mutant seed (Figures 5 and 6). To investigate the potential of LIG6 overexpression to enhance seed germination vigour and longevity after priming, we generated a series of *35S:LIG6* transgenic lines with elevated levels of *LIG6* expression. LIG6 overexpression lines exhibited reduced MTG (improved seed vigour) relative to wild type after accelerated ageing for 7 day at 35°C and 83% RH (Figure 7a). Primed seed of *35S:LIG6* transgenic lines additionally exhibited enhanced seed longevity, revealed by accelerated ageing of primed wild type and overexpression lines (Figure 7a,b). The resistance to seed ageing of lines with elevated levels of *LIG6* coincided with a reduced transcriptional DDR (Supporting Information: Figure S12). This further confirms the role of LIG6 in longevity of primed seed and indicates the potential for using genetic approaches to improve seed longevity after priming treatments.

## 4 Discussion

Germination performance and seedling establishment are routinely improved in many crop species by priming pretreatment of seeds, in which controlled hydration activates pre‐germinative processes without completion of germination. However, seed longevity is reduced in primed seeds of many species, although the molecular basis for this remains obscure (Pagano et al. 2023). Here, RNAseq analysis of naturally aged and osmoprimed Arabidopsis wild type and *lig6lig4* mutant seed lots revealed a transcriptome shift in aged seeds upon imbibition that was largely reversed by seed priming, leading to an imbibition transcriptome similar to their unaged counterparts. Furthermore, this study establishes the importance of DSB repair to these processes, with activation of the transcriptional response to chromosomal breaks in wild type and *lig6lig4* mutant seed, together with increased PCD in the primed DSB repair mutants. This is indicative of DSBs arising during priming, consistent with genome damage being a causative factor of the reduced longevity of primed seeds. Moreover, transgenic lines with enhanced expression of *LIG6* showed increased resistance to accelerated ageing of primed seed. Collectively this data provides the molecular basis for the reduced longevity of primed seed, indicating that loss of longevity in primed seeds is linked to exacerbation of DSBs in primed seed and establishing the genetic requirement for DNA ligases to promote seed viability.

The genetic factors that underlie seed longevity remain incompletely understood, despite the fundamental importance to world agriculture and international germplasm conservation in seed banks (Macovei et al. 2017). Seed vigour and longevity are complex agronomic traits, integrating genetic, biochemical, biophysical and environmental factors including hormone signalling, anti‐oxidant systems, cell wall permeability and maintenance of biological macromolecules (Fleming et al. 2019; Zinsmeister et al. 2020; Arif et al. 2022). Genetic approaches in Arabidopsis and other plants have identified a number of processes that contribute to seed longevity including factors that protect the major cellular macromolecules, including proteins, nucleic acids and membranes (Sano et al. 2016; Zinsmeister et al. 2020; Waterworth et al. 2024). Here we show that seed ageing under ambient conditions is accompanied by the transcriptional induction of GST factors, as observed in a number of environmental stress responses and previously shown to be important for seed viability. GSTs are a large gene family in plants and animals, with specific sub‐families found in vascular plants including tau and phi GSTs and have a range of different substrates and activities. These include detoxification (e.g., herbicides) and glutathione peroxidase activity to reduce organic hydroperoxides of fatty acids and nucleic acids to the corresponding monohydroxyalcohols, conferring oxidative‐stress tolerance (Labrou et al. 2015). GSTs may also function in stress tolerance through a role in cell signalling and some GSTs are highly induced by oxidative stress. Previously, functional roles for GSTU7 were found in seeds, where mutants displayed loss of viability under conditions of oxidative stress (Wu et al. 2020), indicating essential roles of GSTs in supporting seed germination.

Aged seeds also displayed increased expression of hypoxia related genes. A low oxygen response in imbibed seeds was previously reported in *Arabidopsis* (Liew et al. 2024), with a greater response observed in aged seeds, dependent on the WHIRLY1 and WHIRLY3 transcription factors (Taylor et al. 2023). Oxygen levels are sensed by the N‐end rule pathway, whereby oxidation of the amino‐terminal cysteine of group VII Ethylene Response Factor transcription factors (ERFVIIs) results in their degradation in a pathway mediated by ARGINYL TRANSFERASES (ATEs) and the E3 ligase PROTEOLYSIS 6 (PRT6). This pathway can also mediate abiotic stress signalling by NO, which, like oxygen, can react with the ERFVII N‐terminus (Vicente et al. 2017). Plants lacking PRT6 or ATEs have stabilised ERFVII and a constitutive hypoxia response (Gibbs et al. 2011) including elevated expression of 201 genes in the mutants, 45 of which have the hypoxia‐response GO term GO:0071456. Surprisingly, analysis of the expression of these 45 N‐end rule hypoxia genes in seeds found varied expression across the treatments, some displaying high expression in aged imbibed seeds, imbibed seeds or dry seeds (Supporting Information: Figure S13). This indicates that the expression of ERFVII‐hypoxia responsive genes is differentially regulated in response to the different stresses incurred in seeds and may not simply reflect a state of hypoxia. Seed ageing also resulted in increased genome stress, revealed by the increased PCD in *lig6lig4* mutants (Figure 6).

Primed *lig6lig4* DSB repair mutants display significant hypersensitivity to ageing and exhibit an increased transcriptional DDR and PCD after priming, whilst overexpression of LIG6 confers resistance to ageing to primed seed. Taken together this is consistent with priming exacerbating the formation of chromosomal breaks, resulting in reduced longevity which is mitigated by the activity of DNA ligases in wild type seeds. However, priming has beneficial effects on germination vigour in the short term. Our transcriptomic analysis of naturally aged wild type and *lig6lig4* mutants seed revealed that priming reduced ageing‐associated transcript levels, and gene expression profiles of primed seeds displayed greater similarity to those of unaged imbibed seeds. This is in line with activation of pre‐germinative metabolism during priming, reversing the cellular effects of ageing and provides an explanation at the transcriptional level for the improvement germination vigour observed in primed crop species. However, gene expression during imbibition of primed and control seeds displayed key differences, for example in the expression of proteasome factors, indicative of high levels of protein turnover in primed seeds. Furthermore, a large ATM‐dependent transcriptional DNA damage response was observed in primed seeds, both in the primed seed after drying back and upon re‐imbibition, indicative that high levels of chromosomal breaks associated with priming. This observation is further supported by an increase in PCD in the root apical meristem of primed *lig6lig4* seeds post‐germination. Current understanding of the molecular basis of the associated loss of seed longevity remains limited and the increased genome stress was surprising, given that restoration of genome integrity by repair processes is observed during priming as shown in leek (*Allium porrum* L.) seeds (Ashraf and Bray 1993; vanPijlen et al. 1996), with nuclear replicative DNA synthesis and cell cycle progression occurring post‐priming. Repair activities would be expected to reduce genome stress responses in primed seeds, in contrast to the findings of our RNAseq, qPCR and PCD analysis. Our results extend previous reports demonstrating increased expression of base excision repair factors in primed and over‐primed seeds (Forti et al. 2020; Pagano et al. 2022; Pagano et al. 2023). We show that priming activates the canonical transcriptional DDR, previously demonstrated to be a product of ATM‐SOG1 signalling in response to DSBs (Culligan et al. 2006). The increased genome stress, indicative of DSBs in primed seeds, may arise from partial loss of desiccation tolerance, for example through activation of DNA replication origins and initiation of S‐phase, or loss of genome protection factors, as found in some other desiccation tolerant species (Karssen et al. 1990; Chavez et al. 2019). During priming cells of the root tip of tomato embryos progress from the G1 phase of the cell cycle into G2 via a round of replicative DNA synthesis but cell division is not observed (Bino et al. 1992; DawidowiczGrzegorzewska 1997; de Castro et al. 2000). This is consistent with cell cycle activity contributing to the advancement of germination in primed seeds, but potentially leading increased levels of genome stress and contributing to the reduced longevity observed in many species (Fabrissin et al. 2021a). Priming therefore leads to an increased requirement for DNA repair activities in the recovery from extensive genome damage incurred by desiccation and seed ageing (Waterworth et al. 2010). Furthermore, we show increased proteasome transcript levels in primed seeds. The proteasome regulates cellular signalling, including ABA pathways through turnover of ABI5. Reduced ABA signalling in primed seeds has the potential to reduce any residual dormancy but also decrease desiccation tolerance and longevity, given ABA's central role in protecting seeds during maturation drying (Zinsmeister et al. 2020).

The advancement of germination conferred by priming may also arise through storage protein mobilisation, endosperm weakening and DNA repair synthesis, as identified in a number of priming studies (Capron et al. 2007; Waterworth et al. 2015). In addition, stress responses have been associated with vigour improvement in primed seeds, including upregulation of heat shock factors in tomato (Barbosa Batista et al. 2020), and more recently an attenuation of stress responses in Arabidopsis (Gran et al. 2025). The advancement of germination conferred by hydropriming results in enhanced translation capacity as stored mRNAs are recruited to ribosomes, revealed by polysome profiling (Gran et al. 2025). In natural ecosystems in temperate wet climates, seeds of wild species can exist in the soil seed bank for several years, undergoing multiple wet‐dry cycles. Priming‐like activities may therefore effectively occur in the soil seed bank and lead to a requirement for DNA repair pathways to prolong seed longevity (Sano 2025). Seed priming provides a low‐cost, low technology approach which can improve crop yields through improved germination and seedling vigour without a requirement for genetic improvement by plant breeding or gene editing approaches. As such, optimisation of seed priming represents a powerful approach to improve crop yields and stress resistance under changing climates.

## Conflicts of Interest

The authors declare no conflicts of interest.

## Supporting information


**Figure S1.** Frequencies of abnormal seedlings germinated from unaged, primed and aged primed WT and DNA repair mutant seed (as Figure 1a). **Figure S2.** Gene ontology analysis: increased in wild type dry aged seeds vs wild type dry seeds. **Figure S3.** Gene ontology analysis: decreased in wild type dry aged seeds vs wild type dry seeds. **Figure S4.** Gene ontology analysis: increased in wild type imbibed seeds vs wild type dry seeds. **Figure S5.** Gene ontology analysis: increased in wild type aged imbibed seeds vs wild type imbibed seeds. **Figure S6.** Gene ontology analysis: decreased in wild type imbibed seeds vs wild type dry seeds. **Figure S7.** Gene ontology analysis: decreased in wild type aged imbibed seeds vs wild type imbibed seeds. **Figure S8.** Gene ontology analysis: increased in wild type aged imbibed seeds vs wild type imbibed seeds. **Figure S9.** Gene ontology analysis: decreased in wild type aged imbibed seeds vs wild type imbibed seeds. **Figure S10.** Gene ontology analysis: greatest increase in wild type aged imbibed seeds. **Figure S11.** Gene ontology analysis: greatest decrease in wild type aged imbibed seeds. **Figure S12.** DDR gene expression in *DNA LIGASE 6* overexpression lines. **Figure S13** Heatmap of hypoxia related genes.

**Table S1.** Primers.

**Table S2.** GSEA gene lists.

**Table S3.** Differentially expressed genes identified by DeSeq2: wild type dry seeds vs wild type aged dry seeds.

**Table S4.** Differentially expressed genes identified by DeSeq2: wild type dry seeds vs wild type imbibed seeds.

**Table S5.** Differentially expressed genes identified by DeSeq2: wild type aged dry seeds vs wild type aged imbibed seeds.

**Table S6.** Differentially expressed genes identified by DeSeq2: wild type imbibed seeds vs wild type aged imbibed seeds.

**Table S7.** Differentially expressed genes identified by DeSeq2: wild type dry seeds vs wild type aged primed dry seeds.

**Table S8.** Differentially expressed genes identified by DeSeq2: wild type dry seeds vs wild type aged primed imbibed seeds.

**Table S9.** Differentially expressed genes identified by DeSeq2: wild type dry seeds vs *lig6lig4* dry seeds.

**Table S10.** Differentially expressed genes identified by DeSeq2: wild type aged dry seeds vs *lig6lig4* aged dry seeds.

**Table S11.** Differentially expressed genes identified by DeSeq2: wild type aged imbibed seeds vs *lig6lig4* aged imbibed seeds.

**Table S12.** Differentially expressed genes identified by DeSeq2: wild type primed dry seeds vs *lig6lig4* primed dry seeds.

**Table S13.** Differentially expressed genes identified by DeSeq2: wild type primed imbibed seeds vs *lig6lig4* primed imbibed seeds.

supmat.

## Data Availability

The data that support the findings of this study are openly available in National Center for Biotechnology (NCBI) Information Sequence Read Archive (SRA) at https://www.ncbi.nlm.nih.gov/sra, reference number PRJNA875594.
