# Supplementary material for: DNA Double Strand Break Repair Is Important for the Longevity of Primed Seeds
Source: Plant Cell Environ. 2025 Aug 21;48(12):8469–82. doi: 10.1111/pce.70142 (PMC12586901; doi:10.1111/pce.70142)
Supplement: Supplementary file 1 — Figure S1. Frequencies of abnormal seedlings germinated from unaged, primed and aged primed WT and DNA repair mutant seed (as Figure 1a). Figure S2. Gene ontology analysis: increased in wild type dry aged seeds vs wild type dry seeds. Figure S3. Gene ontology analysis: decreased in wild type dry aged seeds vs wild type dry seeds. Figure S4. Gene ontology analysis: increased in wild type imbibed seeds vs wild type dry seeds. Figure S5. Gene ontology analysis: increased in wild type aged imbibed seeds vs wild type imbibed seeds. Figure S6. Gene ontology analysis: decreased in wild type imbibed seeds vs wild type dry seeds. Figure S7. Gene ontology analysis: decreased in wild type aged imbibed seeds vs wild type imbibed seeds. Figure S8. Gene ontology analysis: increased in wild type aged imbibed seeds vs wild type imbibed seeds. Figure S9. Gene ontology analysis: decreased in wild type aged imbibed seeds vs wild type imbibed seeds. Figure S10. Gene ontology analysis: greatest increase in wild type aged imbibed seeds. Figure S11. Gene ontology analysis: greatest decrease in wild type aged imbibed seeds. Figure S12. DDR gene expression in DNA LIGASE 6 overexpression lines. Figure S13 Heatmap of hypoxia related genes. [file PCE-48-8469-s010.docx]

## Supporting Information

Article title: DNA double strand break repair is important for the longevity of primed seeds

Authors: Wanda M Waterworth^1^*, Dapeng Wang^2^*, Lerissa S Dsilva^1^ and Christopher E West^1^

Article acceptance date: Click here to enter a date.

The following Supporting Information is available for this article:

**Fig. S1** Frequencies of abnormal seedlings germinated from unaged, primed and aged primed WT and DNA repair mutant seed (as Figure 1a). Seeds of WT and DNA repair mutant lines were primed for 48h with -0.75 MPa PEG6000 before drying. Frequency of abnormal seedlings from primed Col-0 and mutant primed seed was analysed before and after accelerated aging at 35 °C and 83% RH for 7 days. Seeds were stratified at 4 °C for 2 d before transfer to 23 °C 16-h day and seedling abnormalities were analysed in accordance with ISTA guidelines in germinated seedlings at 8 days post-stratification. Germination data of seed lots analysed is presented in Figure 1a. Error bars represent the SEM of the mean of three replicates of 30 seedlings.


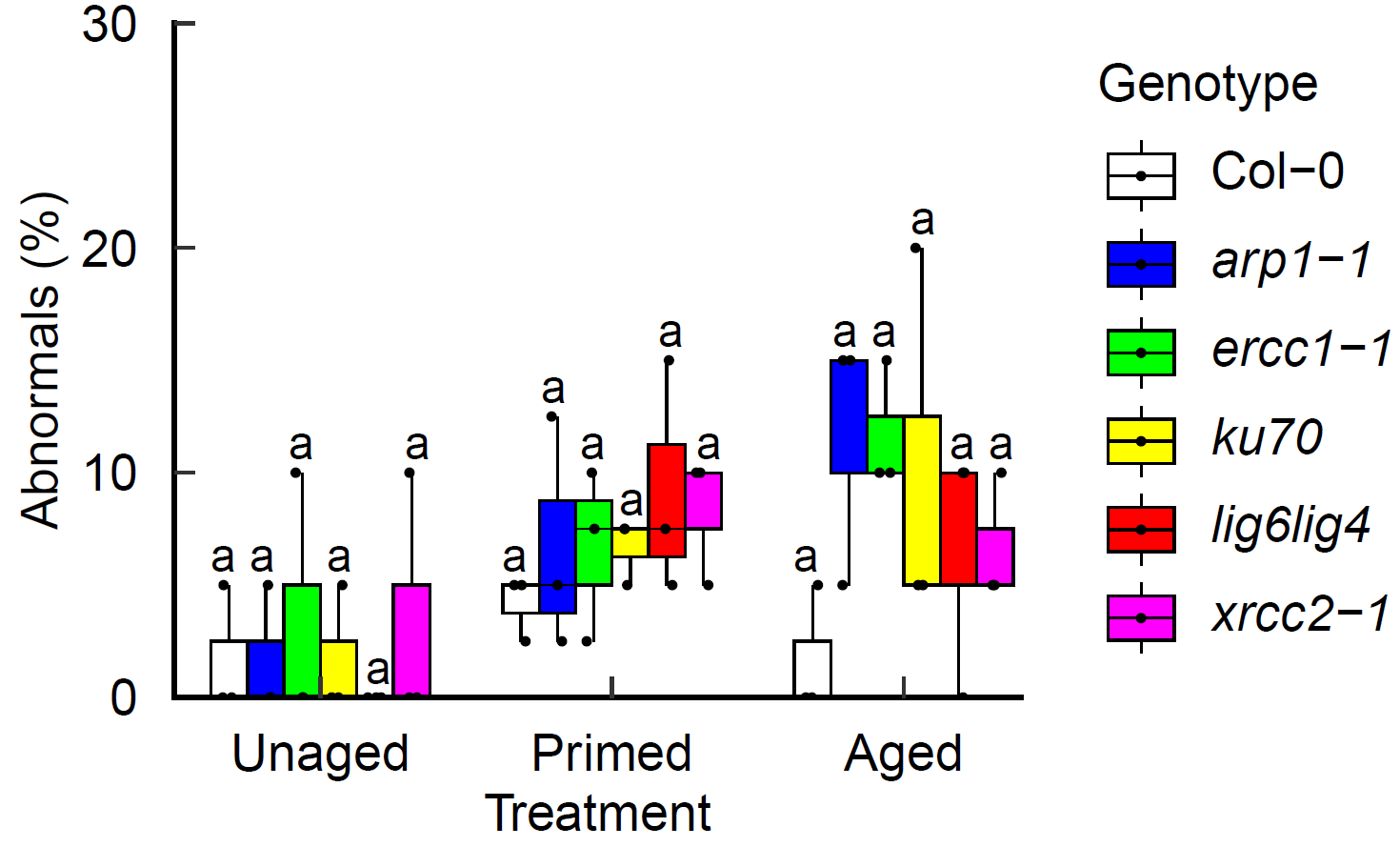


**Fig. S2** Gene ontology analysis: increased in wild type dry aged seeds vs wild type dry seeds

**
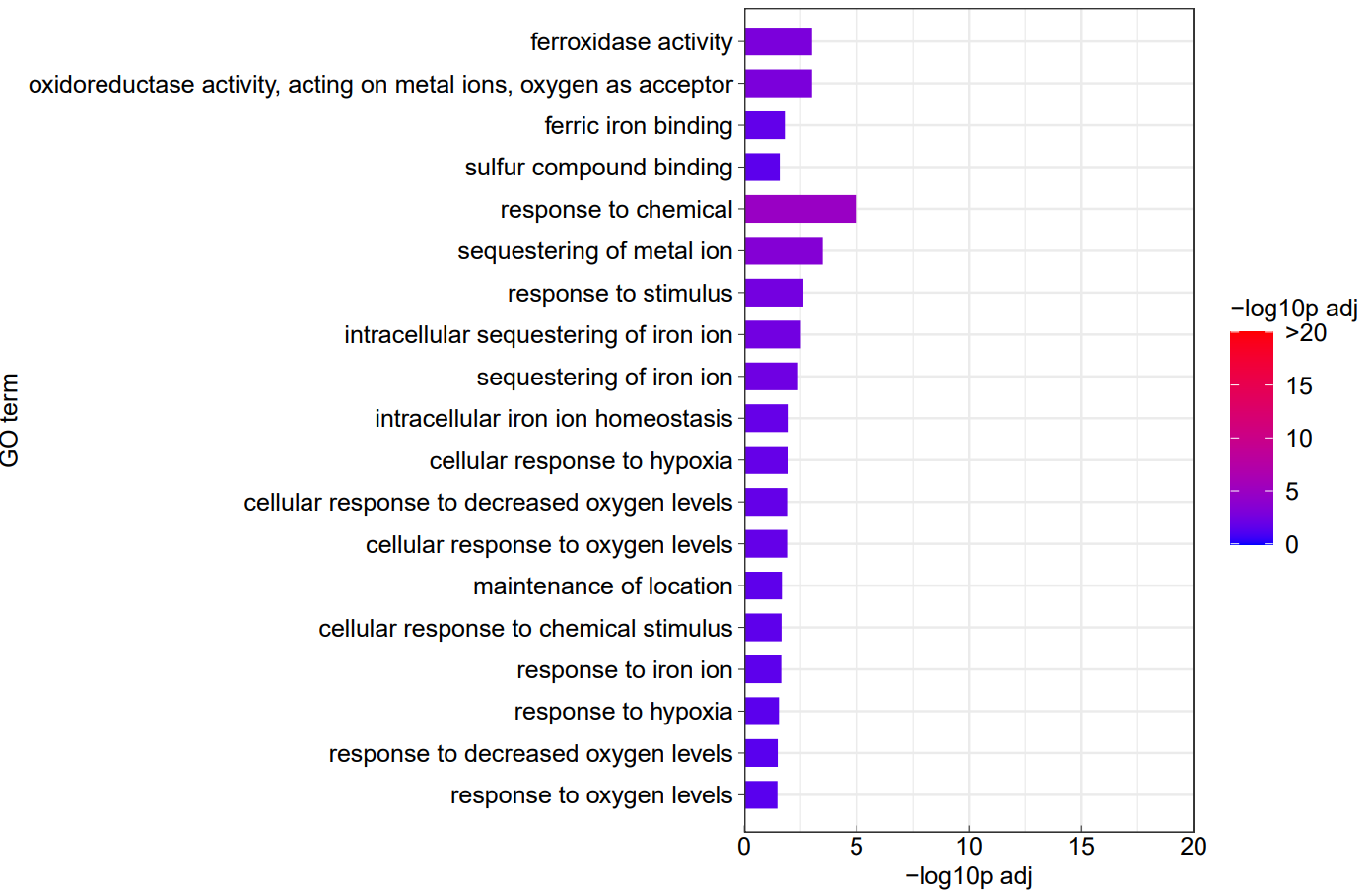
**

**Fig. S3** Gene ontology analysis: decreased in wild type dry aged seeds vs wild type dry seeds


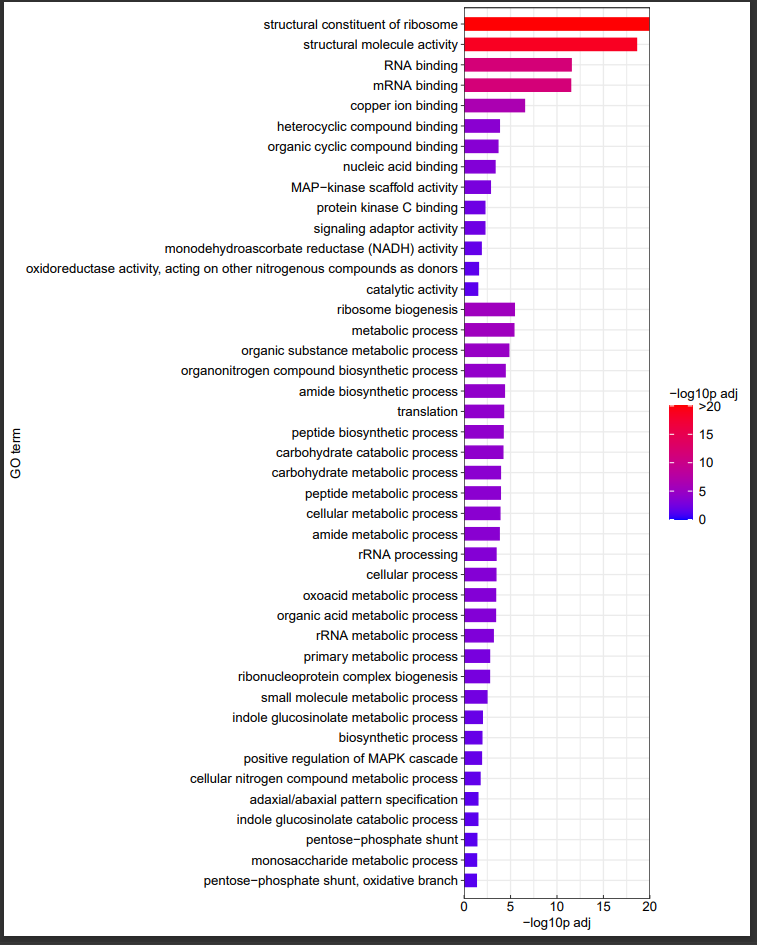


**Fig. S4** Gene ontology analysis: increased in wild type imbibed seeds vs wild type dry seeds


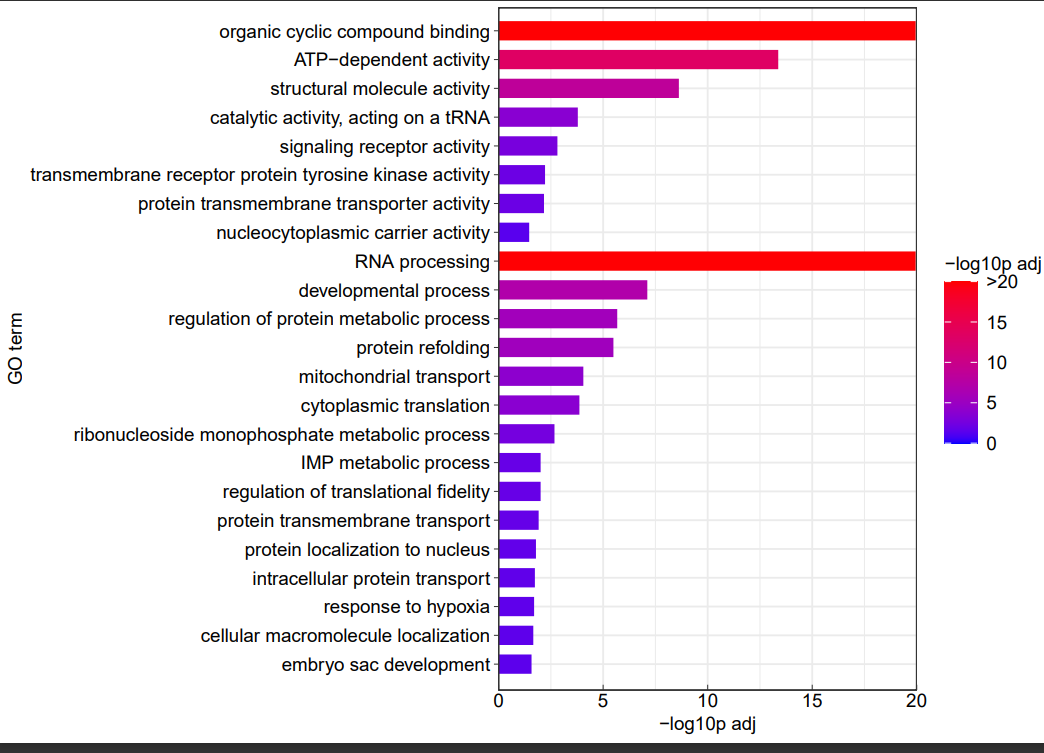


**Fig. S5** Gene ontology analysis: increased in wild type aged imbibed seeds vs wild type imbibed seeds


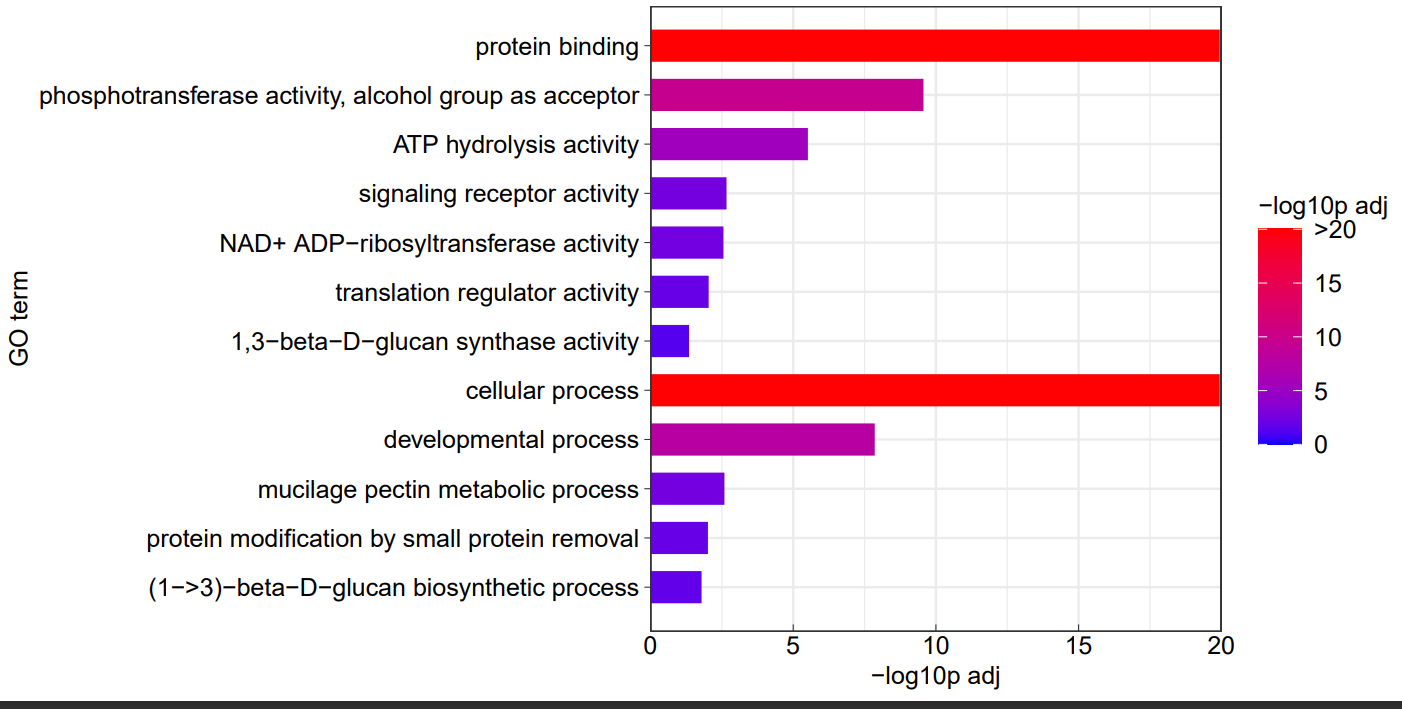


**Fig. S6** Gene ontology analysis: decreased in wild type imbibed seeds vs wild type dry seeds

**
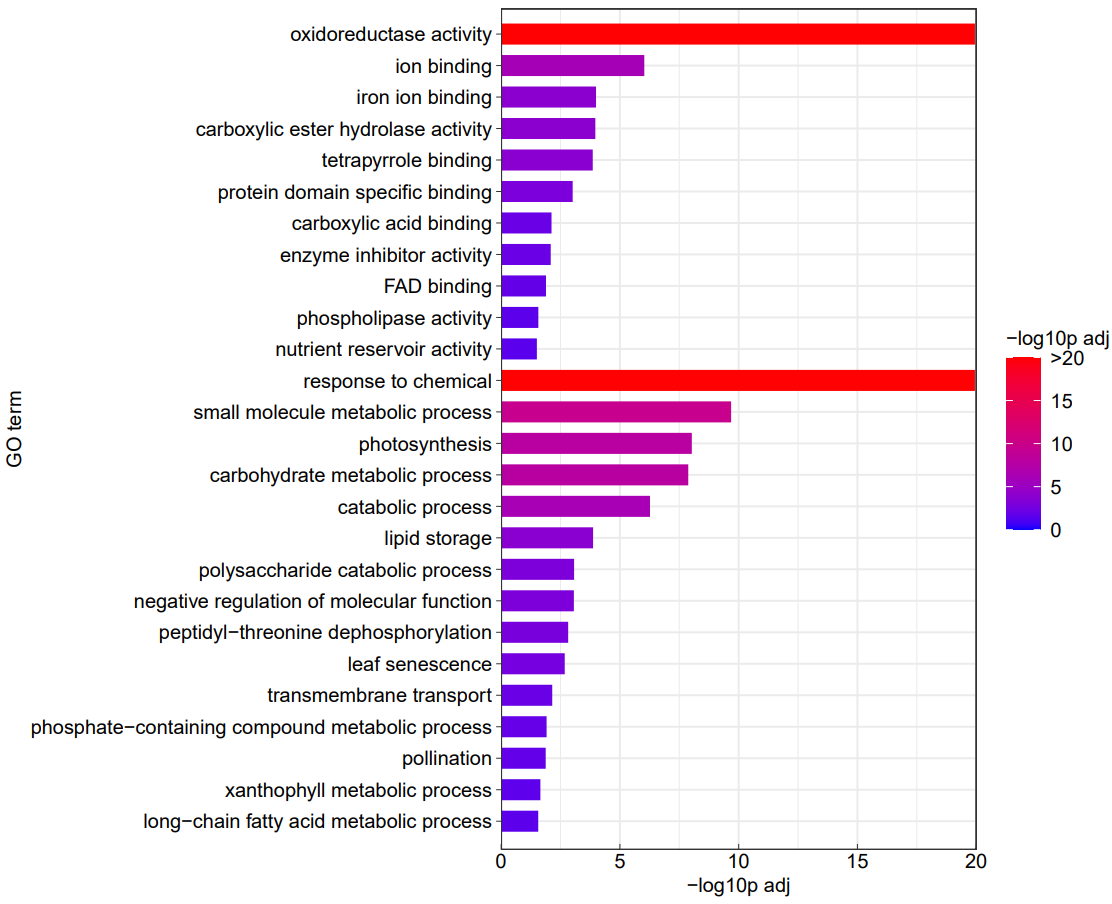
**

**Fig. S7** Gene ontology analysis: decreased in wild type aged imbibed seeds vs wild type imbibed seeds

**
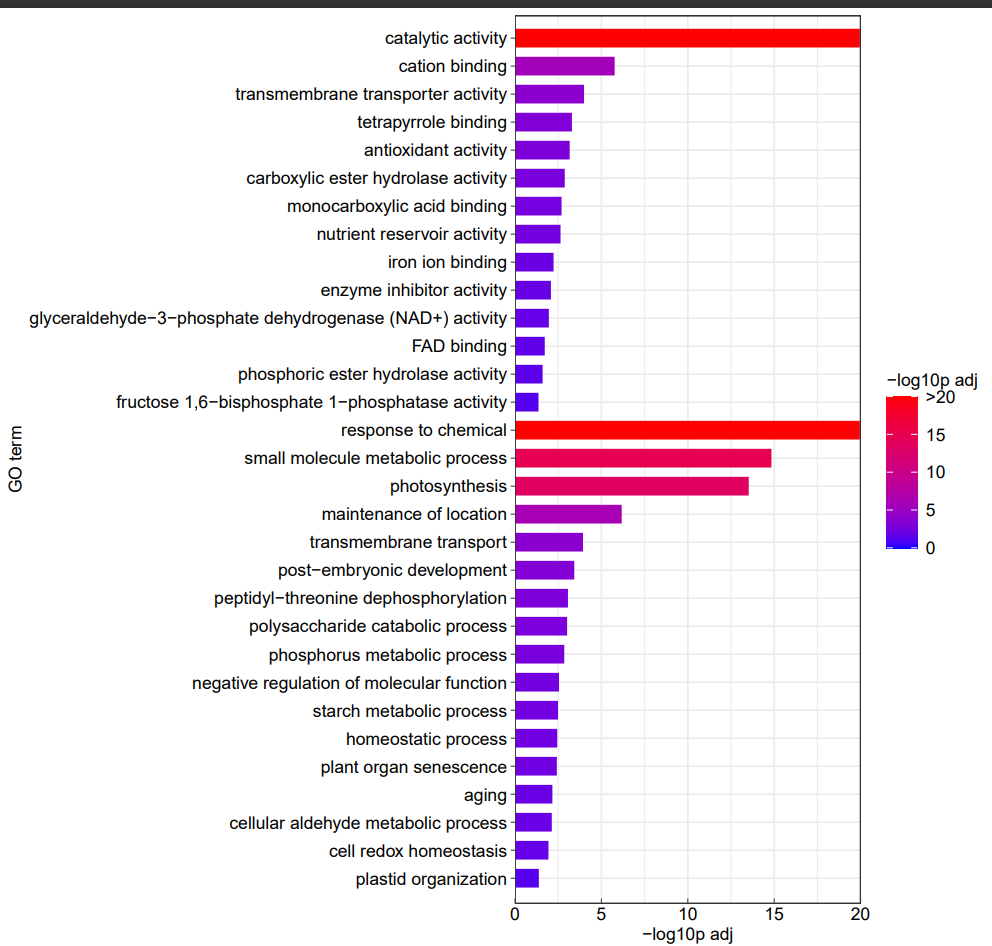
**

**Fig. S8** Gene ontology analysis: increased in wild type aged imbibed seeds vs wild type imbibed seeds


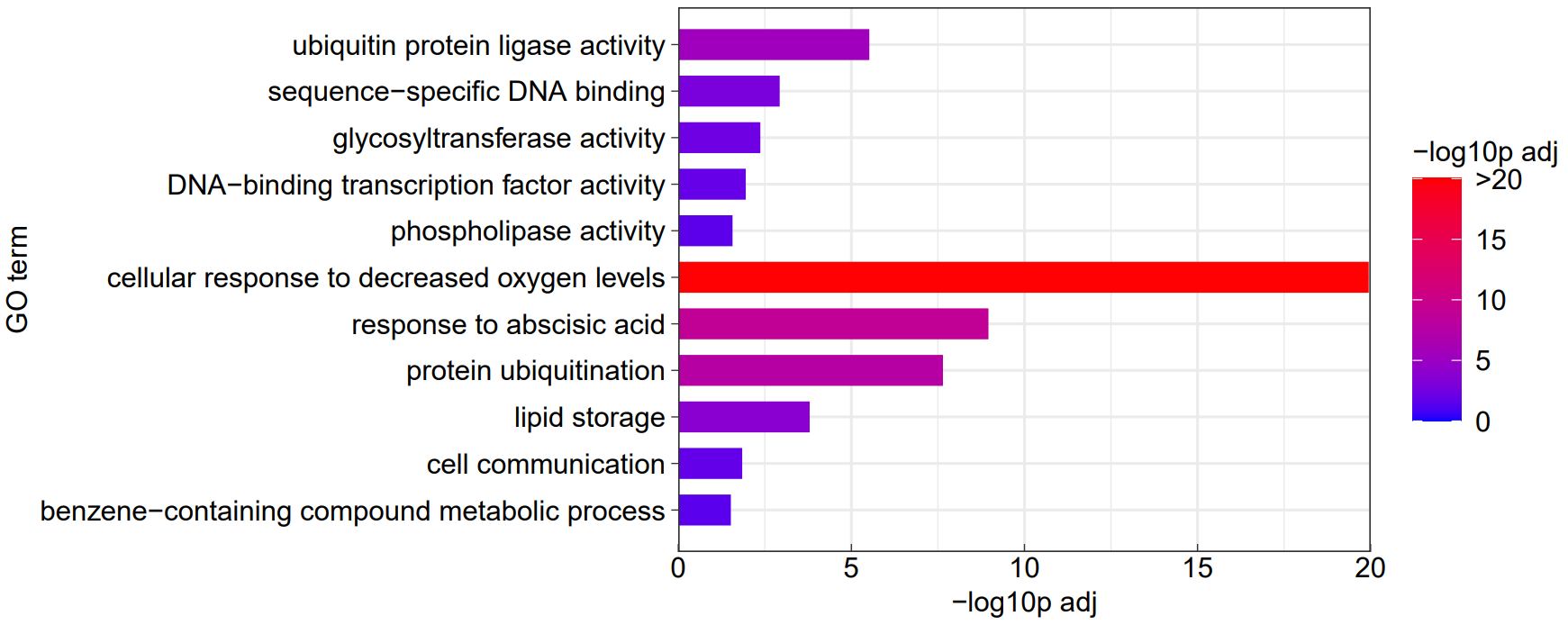


**Fig. S9** Gene ontology analysis: decreased in wild type aged imbibed seeds vs wild type imbibed seeds


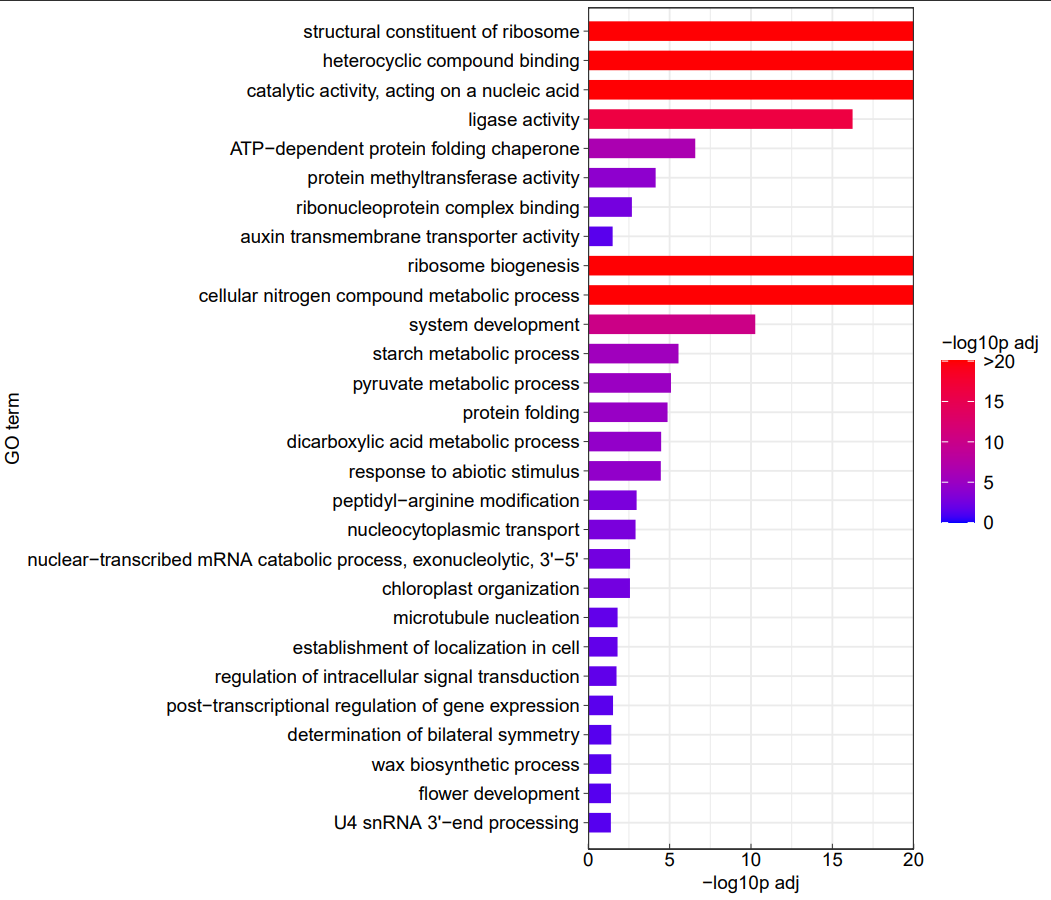


**Fig. S10** Gene ontology analysis: greatest increase in wild type aged imbibed seeds


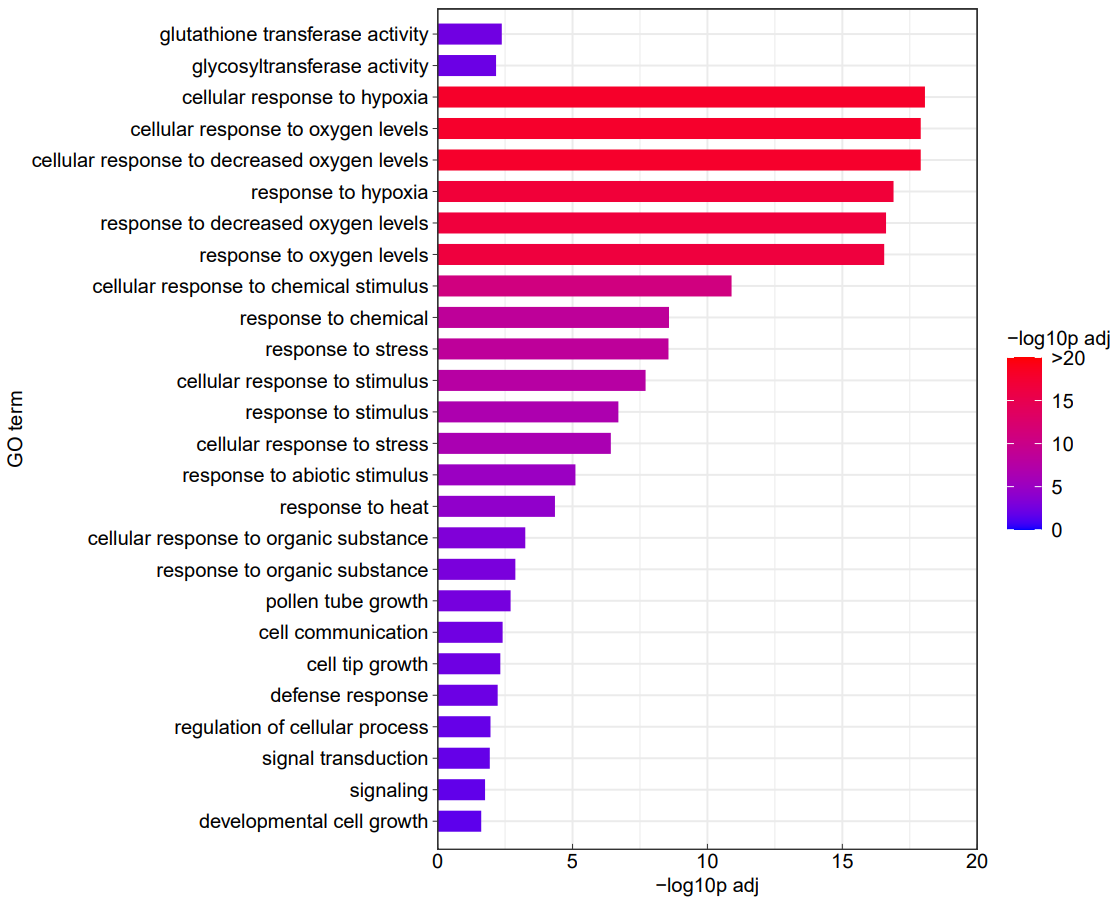


**Fig. S11** Gene ontology analysis: greatest decrease in wild type aged imbibed seeds


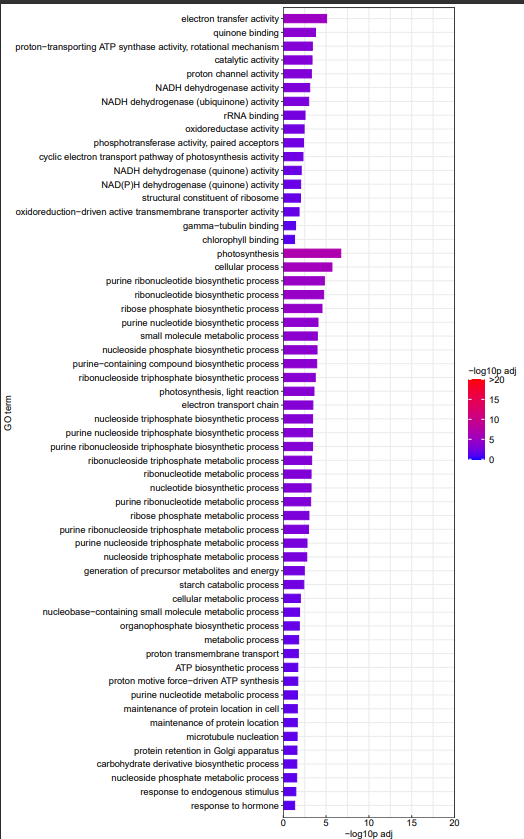


**Fig. S12** DDR gene expression in *DNA LIGASE 6* overexpression lines. Germination performance of transgenic *35S:LIG6* lines of Arabidopsis with increased expression of *LIG6* relative to wild type lines is presented in Figure 7. The transcriptional DDR, indicative of genome stress, is lower in seed of *DNA LIGASE 6* overexpression lines relative to Col-0 after seed priming. qPCR analysis of (a) *RAD51* and (b) *XRI1* expression in 0h (dry) or 6h (imbibed) seeds*.* Letters denote homogeneous subsets (p<0.01, ANOVA with Tukey correction).


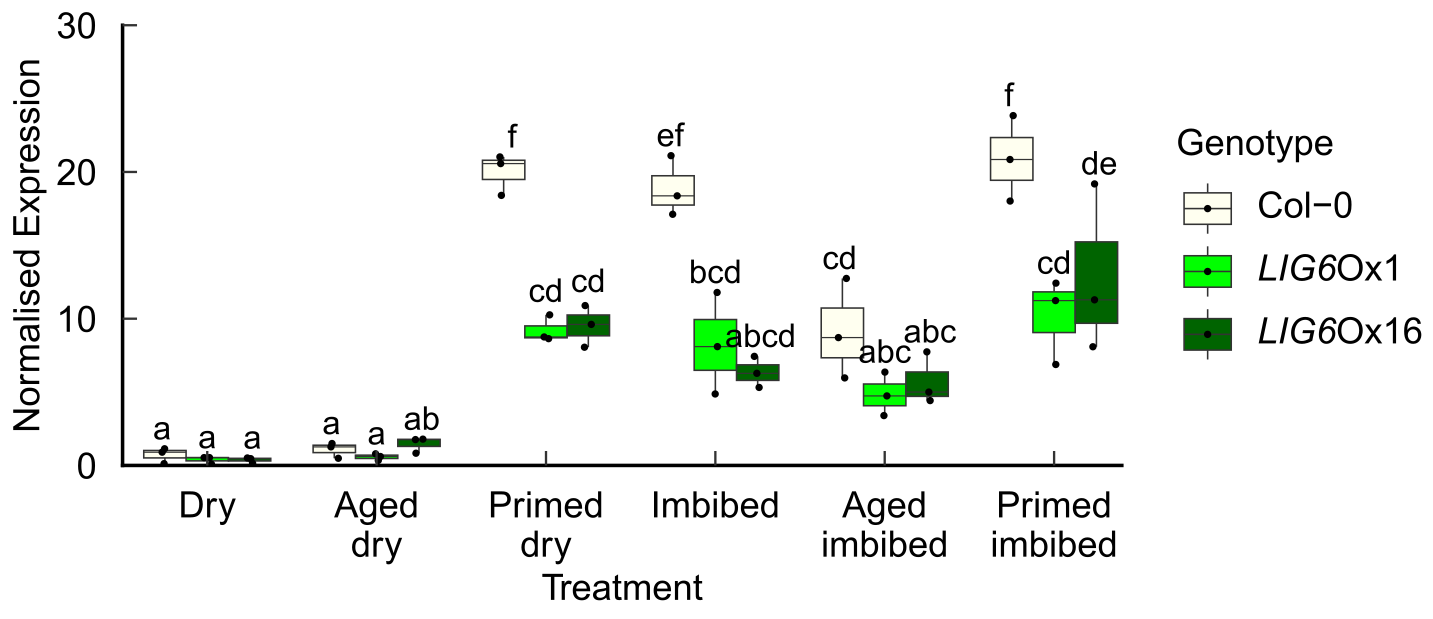
(a)

(b)


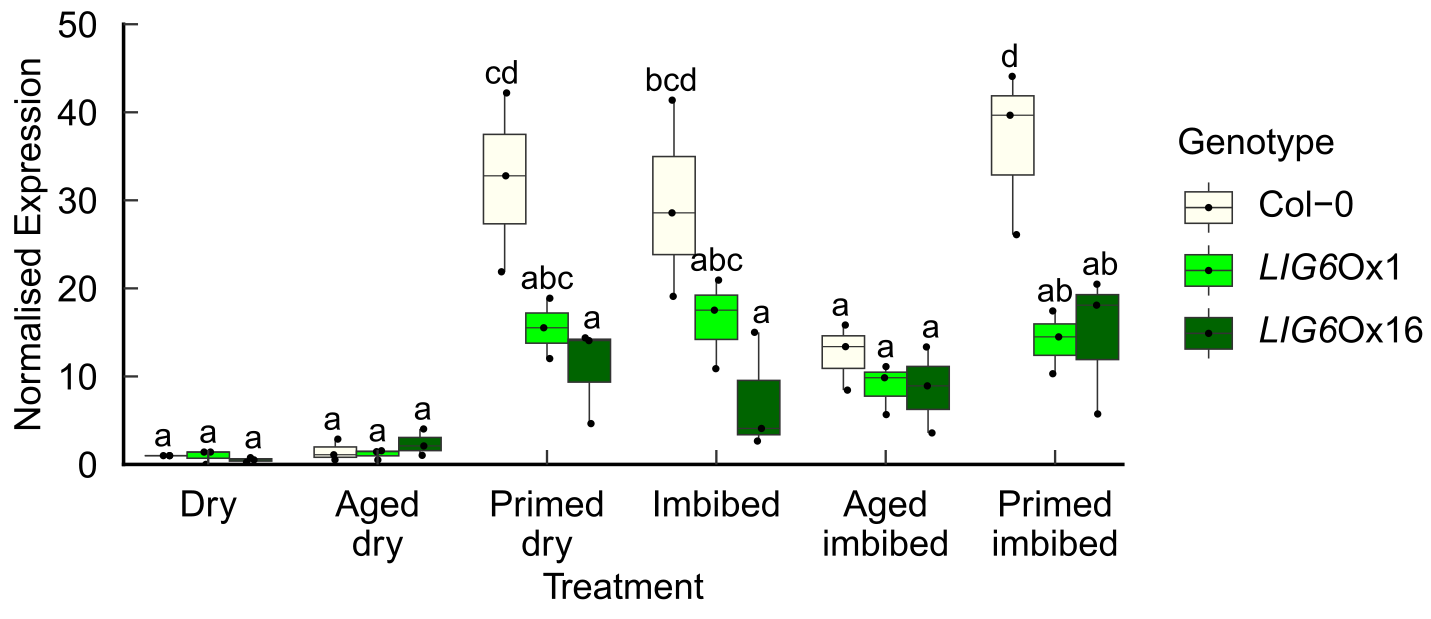


**Fig. S13** Heatmap of hypoxia related genes. Transcript levels across wild type seed samples of the 45 ERFVII responsive genes that have the hypoxia-response GO term GO:0071456


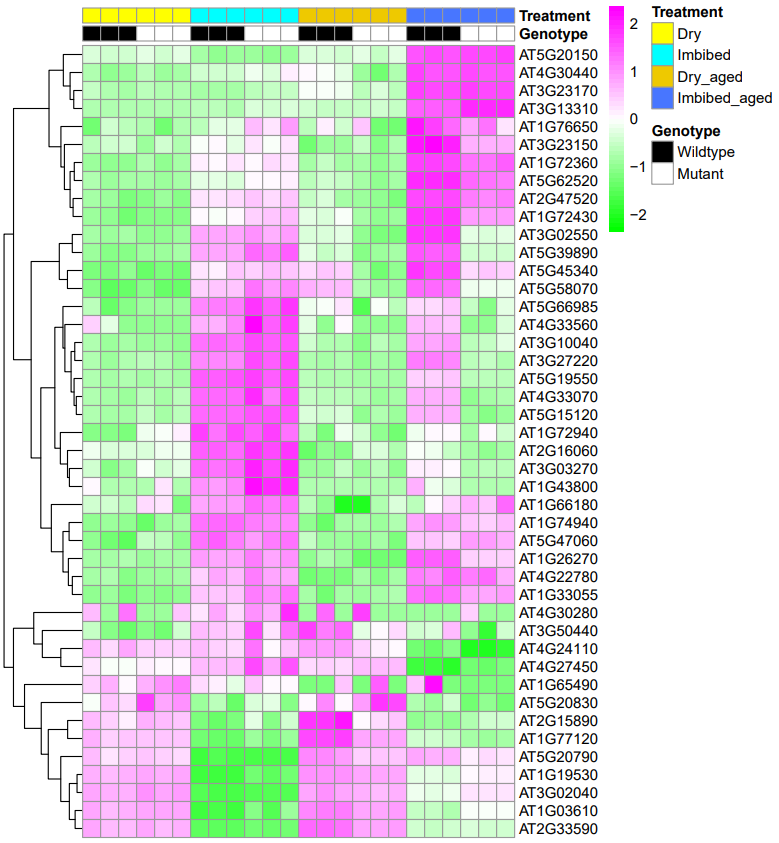


**Table S1 Primers**

**Table S2** GSEA gene lists

Gene set enrichment gene lists (GMX file): Gene sets were identified through analysis of published germination microarray studies (Nakabayashi et al., 2005) and transcriptional responses to gamma irradiation (Culligan et al., 2006).

**Table S3** Differentially expressed genes identified by DeSeq2: wild type dry seeds vs wild type aged dry seeds

**Table S4** Differentially expressed genes identified by DeSeq2: wild type dry seeds vs wild type imbibed seeds

**Table S5** Differentially expressed genes identified by DeSeq2: wild type aged dry seeds vs wild type aged imbibed seeds

**Table S6** Differentially expressed genes identified by DeSeq2: wild type imbibed seeds vs wild type aged imbibed seeds

**Table S7** Differentially expressed genes identified by DeSeq2: wild type dry seeds vs wild type aged primed dry seeds

**Table S8** Differentially expressed genes identified by DeSeq2: wild type dry seeds vs wild type aged primed imbibed seeds

**Table S9** Differentially expressed genes identified by DeSeq2: wild type dry seeds vs lig6lig4 dry seeds

**Table S10** Differentially expressed genes identified by DeSeq2: wild type aged dry seeds vs lig6lig4 aged dry seeds

**Table S11** Differentially expressed genes identified by DeSeq2: wild type aged imbibed seeds vs lig6lig4 aged imbibed seeds

**Table S12** Differentially expressed genes identified by DeSeq2: wild type primed dry seeds vs lig6lig4 primed dry seeds

**Table S13** Differentially expressed genes identified by DeSeq2: wild type primed imbibed seeds vs lig6lig4 primed imbibed seeds
