# Supplementary material for: DNA Double Strand Break Repair Is Important for the Longevity of Primed Seeds
Source: Plant Cell Environ. 2025 Aug 21;48(12):8469–82. doi: 10.1111/pce.70142 (PMC12586901; doi:10.1111/pce.70142)
Supplement: Supplementary file 15 — supmat. [file PCE-48-8469-s002.docx]

## Supporting Information (brief legends)

Table S1: Primers

Table S2: GSEA gene lists

Gene set enrichment gene lists (GMX file): Gene sets were identified through analysis of published germination microarray studies (Nakabayashi *et al.*, 2005) and transcriptional responses to gamma irradiation (Culligan *et al.*, 2006).

Table S3: Differentially expressed genes identified by DeSeq2: wild type dry seeds vs wild type aged dry seeds

Table S4: Differentially expressed genes identified by DeSeq2: wild type dry seeds vs wild type imbibed seeds

Table S5: Differentially expressed genes identified by DeSeq2: wild type aged dry seeds vs wild type aged imbibed seeds

Table S6: Differentially expressed genes identified by DeSeq2: wild type imbibed seeds vs wild type aged imbibed seeds

Table S7: Differentially expressed genes identified by DeSeq2: wild type dry seeds vs wild type aged primed dry seeds

Table S8: Differentially expressed genes identified by DeSeq2: wild type dry seeds vs wild type aged primed imbibed seeds

Table S9: Differentially expressed genes identified by DeSeq2: wild type dry seeds vs *lig6lig4* dry seeds

Table S10: Differentially expressed genes identified by DeSeq2: wild type aged dry seeds vs *lig6lig4* aged dry seeds

Table S11: Differentially expressed genes identified by DeSeq2: wild type aged imbibed seeds vs *lig6lig4* aged imbibed seeds

Table S12: Differentially expressed genes identified by DeSeq2: wild type primed dry seeds vs *lig6lig4* primed dry seeds

Table S13: Differentially expressed genes identified by DeSeq2: wild type primed imbibed seeds vs *lig6lig4* primed imbibed seeds

Figure S1: Frequencies of abnormal seedlings germinated from unaged, primed aged primed WT and DNA repair mutant seed (as Figure 1a). Seeds of WT and DNA repair mutant lines were primed for 48h with -0.75 MPa PEG6000 before drying. Frequency of abnormal seedlings from primed Col-0 and mutant primed seed was analysed before and after accelerated aging at 35 °C and 83% RH for 7 days. Seeds were stratified at 4 °C for 2 d before transfer to 23 °C 16-h day and seedling abnormalities were analysed in accordance with ISTA guidelines in germinated seedlings at 8 days post-stratification. Germination data of seedlots analysed is presented in Figure 1a**.** Error bars represent the SEM of the mean of three replicates of 30 seedlings.

Figure S2: Gene ontology analysis: increased in wild type dry aged seeds vs wild type dry seeds

Figure S3: Gene ontology analysis: decreased in wild type dry aged seeds vs wild type dry seeds

Figure S4: Gene ontology analysis: increased in wild type imbibed seeds vs wild type dry seeds

Figure S5: Gene ontology analysis: increased in wild type aged imbibed seeds vs wild type imbibed seeds

Figure S6: Gene ontology analysis: decreased in wild type imbibed seeds vs wild type dry seeds

Figure S7: Gene ontology analysis: decreased in wild type aged imbibed seeds vs wild type imbibed seeds

Figure S8: Gene ontology analysis: increased in wild type aged imbibed seeds vs wild type imbibed seeds

Figure S9: Gene ontology analysis: decreased in wild type aged imbibed seeds vs wild type imbibed seeds

Figure S10: Gene ontology analysis: greatest increase in wild type aged imbibed seeds

Figure S11: Gene ontology analysis: greatest decrease in wild type aged imbibed seeds

Figure S12: DDR gene expression in *DNA LIGASE 6* overexpression lines. Germination performance of transgenic *35S:LIG6* lines of Arabidopsis with increased expression of *LIG6* relative to wild type lines is presented in Figure 7. The transcriptional DDR, indicative of genome stress, is lower in seed of *DNA LIGASE 6* overexpression lines relative to WT after seed priming. qPCR analysis of (a) *RAD51* and (b) *XRI1 expression* in 0h (dry) or 6h (imbibed) seeds*.* Letters denote homogeneous subsets (p<0.01, ANOVA with Tukey correction).

Figure S13: Heatmap of hypoxia related genes. Transcript levels across wild type seed samples of the 45 ERFVII responsive genes that have the hypoxia-response GO term GO:0071456.
